# Supplementary material for: Expanding N-glycopeptide identifications by modeling fragmentation, elution, and glycome connectivity
Source: Nat Commun. 2024 Jul 22;15:6168. doi: 10.1038/s41467-024-50338-5 (PMC11263600; doi:10.1038/s41467-024-50338-5)
Supplement: Supplementary file 1 — Supplementary Information [file 41467_2024_50338_MOESM1_ESM.pdf]

Expanding *N*-Glycopeptide Identifications By Modeling Fragmentation, Elution, and Glycome Connectivity

Joshua Klein[1], Luis Carvalho[1,2], and Joseph Zaia[1,3]

[1]Program for Bioinformatics, Boston University
[2]Department of Math and Statistics, Boston University
[3]Department of Biochemistry, Boston University

July 7, 2024

Contents

1 Supplementary Figures **2**
1.1 PXD005931 Re-analysis . . . 2
1.2 Gradient Boosting Peptide Intensity Correlation . . . 2
1.3 Peak Intensity Features . . . 4
1.4 Predicted Intensity Correlation By Precursor Charge State . . . 7
1.5 Fragmentation Model Features . . . 9
1.6 Overlap with pGlyco3 . . . 11
1.7 Example of site-specific glycome network smoothing . . . 14
1.8 Yeast Entrapment Study . . . 15
1.9 Intra-cluster Similarity Validation . . . 18
1.10 Predicted Spectra . . . 18
1.11 *N*-Glycopeptide Spectra . . . 18
1.12 *O*-Glycopeptide Spectra . . . 21
1.13 Fitted Retention Time Models . . . 21

2 Supplementary Methods **48**
2.1 Adduct Deconvolution and Retention Time Modeling . . . 48
2.1.1 Initial Adduct Deconvolution . . . 48
2.1.2 Retention Time Modeling . . . 48
2.2 Glycan Composition Fragments and Indexing . . . 52
2.3 Observation Partitioning For Fragmentation Modeling . . . 54
2.4 Fragmentation Modeling Features . . . 54
2.5 Site-Specific Glycome Network Smoothing . . . 55
2.6 Glycome Neighborhood Structure . . . 57
2.7 Run Time . . . 57
2.8 Revision Counts . . . 58

1 Supplementary Figures

1.1 PXD005931 Re-analysis

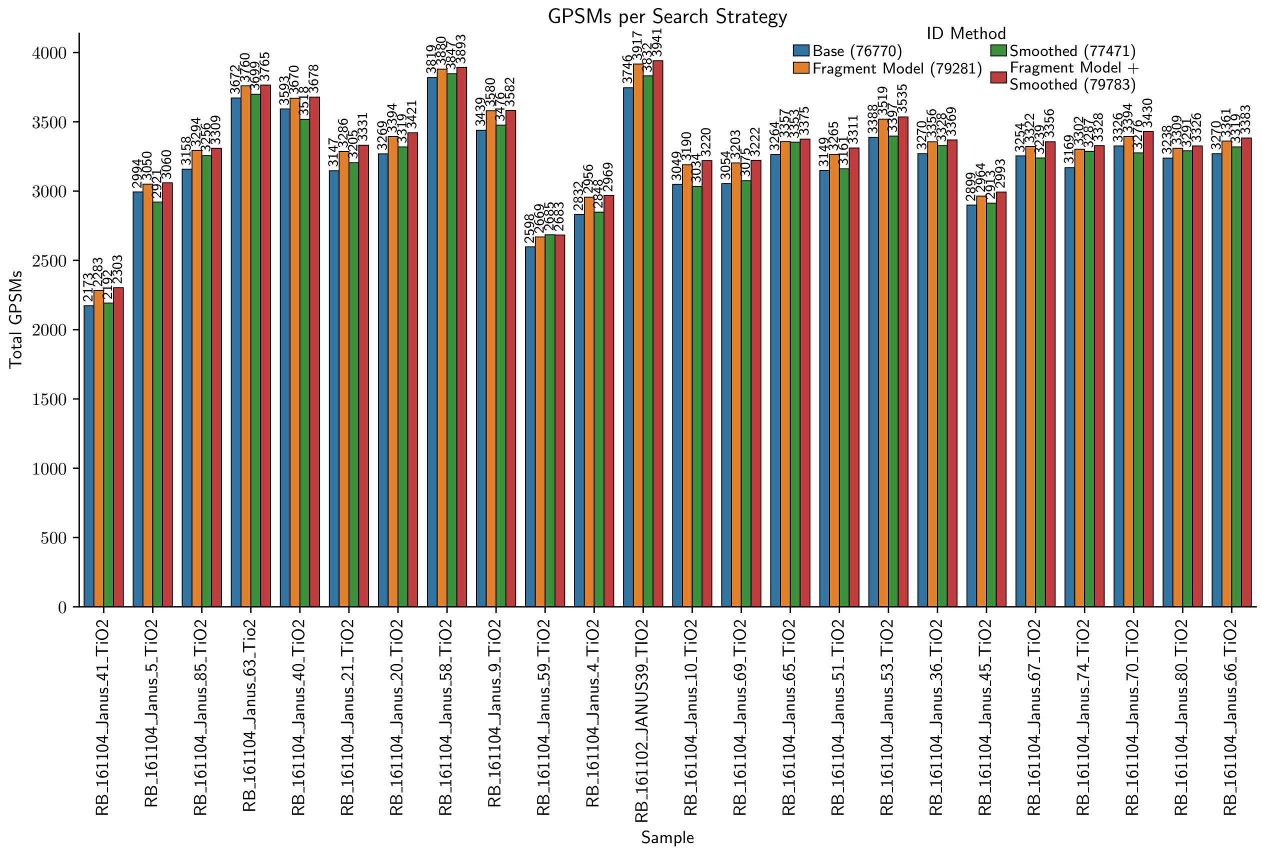

Figure 1: A more granular view of PXD005931 identifications by strategy. The GPSM counts for each sample in PXD005931 for each search strategy.

1.2 Gradient Boosting Peptide Intensity Correlation

We adapted the design of MS^2PIP (Gabriels *et al.*, 2019) to train an ion series and charge state-specific gradient boosting regression model for peptide b and y ions of charge states 1+ and 2+ using the same training and testing spectra used for the mouse tissue dataset. We modified the design to only train and predict on observed product ions. We then predicted peptide fragment intensities for all training and testing spectra and computed the Pearson correlation for each spectrum. The distribution for these correlations are shown in Figure 2.

Although the model performed well on the same dataset it trained on, it did not generalize well on the test dataset. The gradient boosting regression did predict better than the multinomial logistic regression, but its over-fitting makes using this version less reliable.

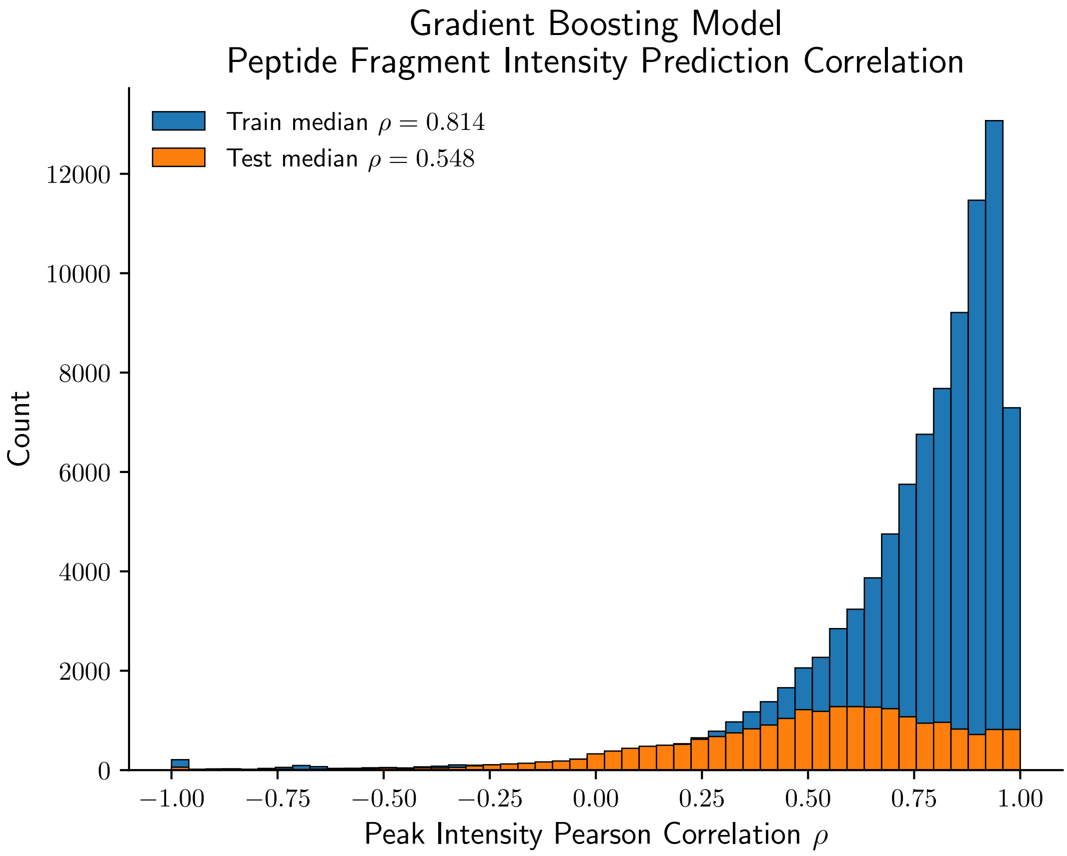

Figure 2: Gradient boosting forest for peptide intensity prediction. The peptide Pearson correlation for the training tissues (Heart, Kidney, Lung, Liver) and test tissue (Brain) listing the median value in the legend.

1.3 Peak Intensity Features

In this section we show the feature weights from the multinomial logistic regression models in each partition for each branch of the model. Feature names shown here use the term to refers to peptide+Y fragments. Parameter significance was calculated using a Wald test, but these statistics are given purely for graphing, all features, regardless of significance, are used for intensity prediction.

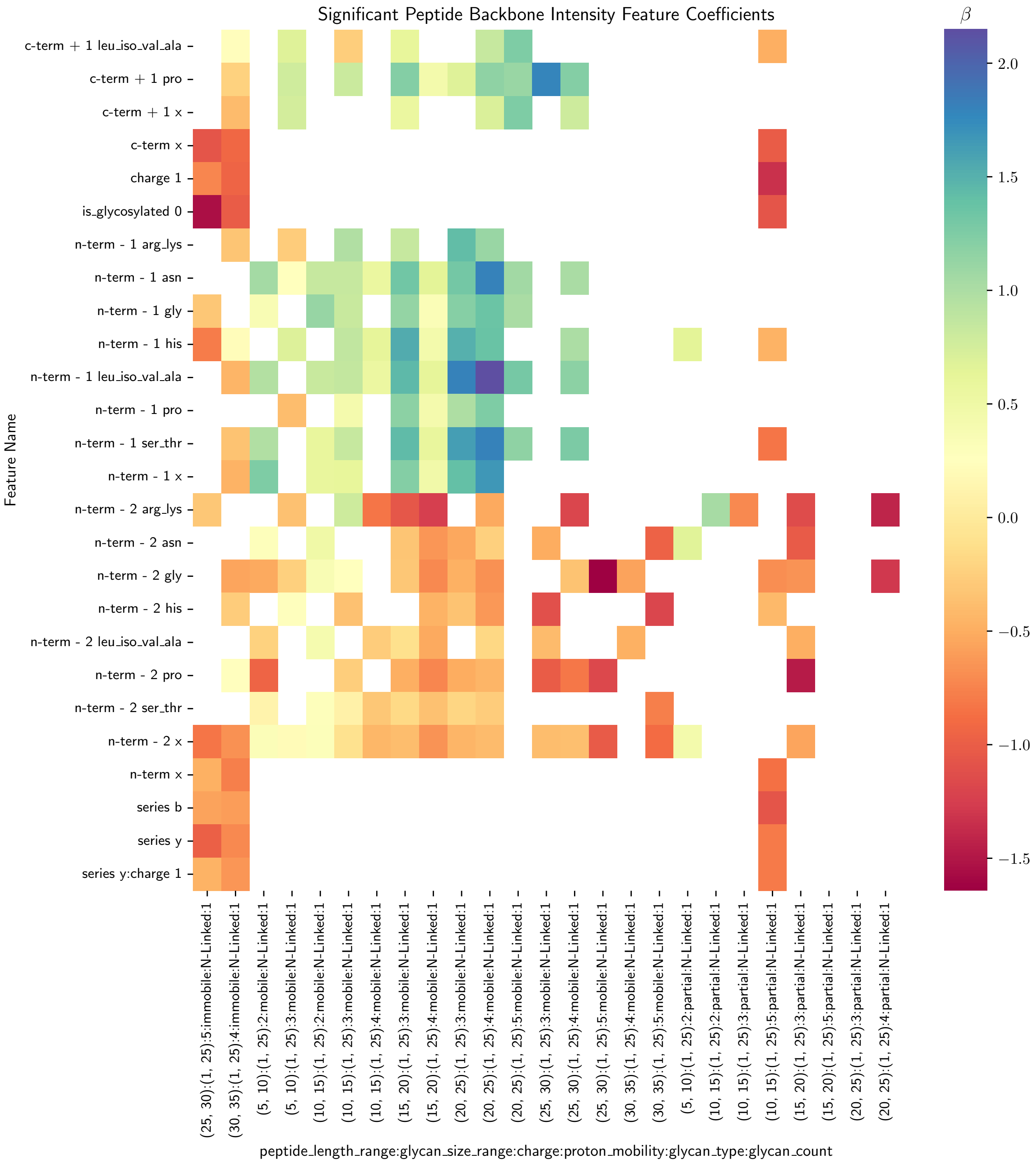

Figure 3: Peptide intensity prediction coefficients. The intensity prediction coefficients for features found statistically significant in at least four partitions for peptide b and y ions.

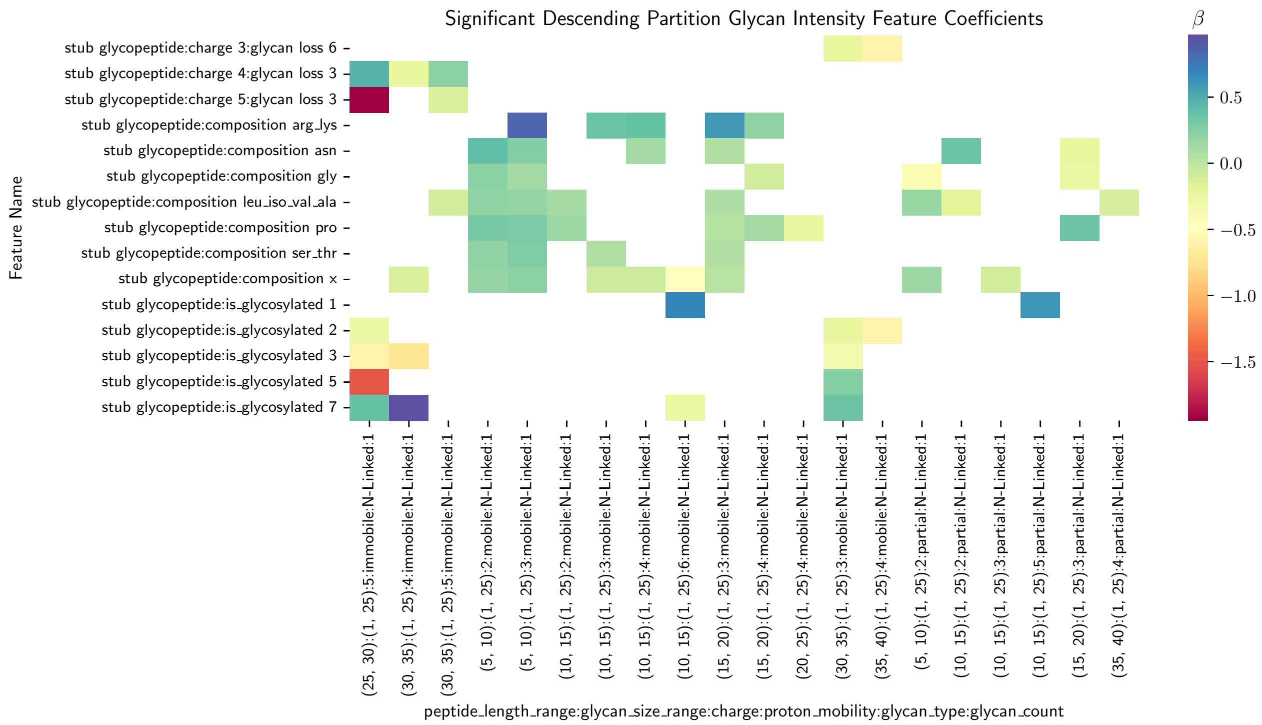

Figure 4: Glycan intensity coefficient predictions for descending intensity. The intensity prediction coefficients for features found statistically significant in at least two partitions for peptide+Y ions where the smallest glycan fragment is the most intense ion in the series.

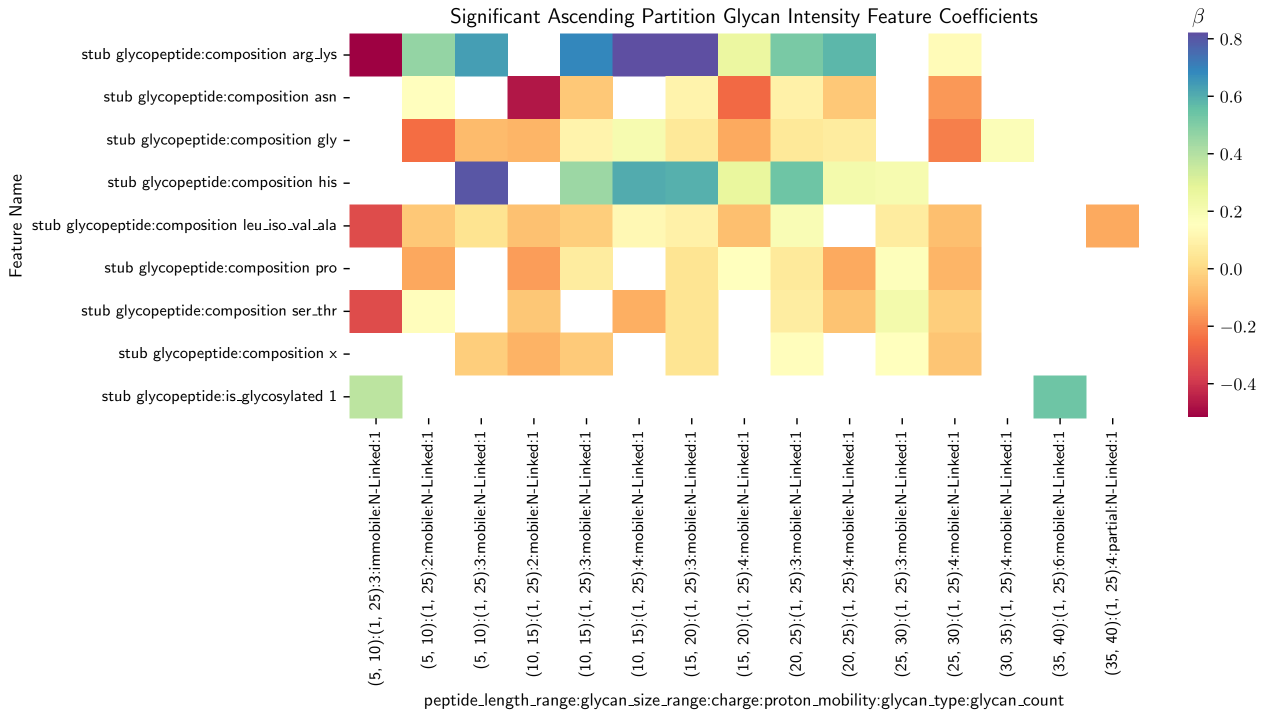

Figure 5: Glycan intensity coefficient predictions for ascending intensity. The intensity prediction coefficients for features found statistically significant in at least two partitions for peptide+Y ions where the smallest glycan fragment is the least intense ion in the series.

1.4 Predicted Intensity Correlation By Precursor Charge State

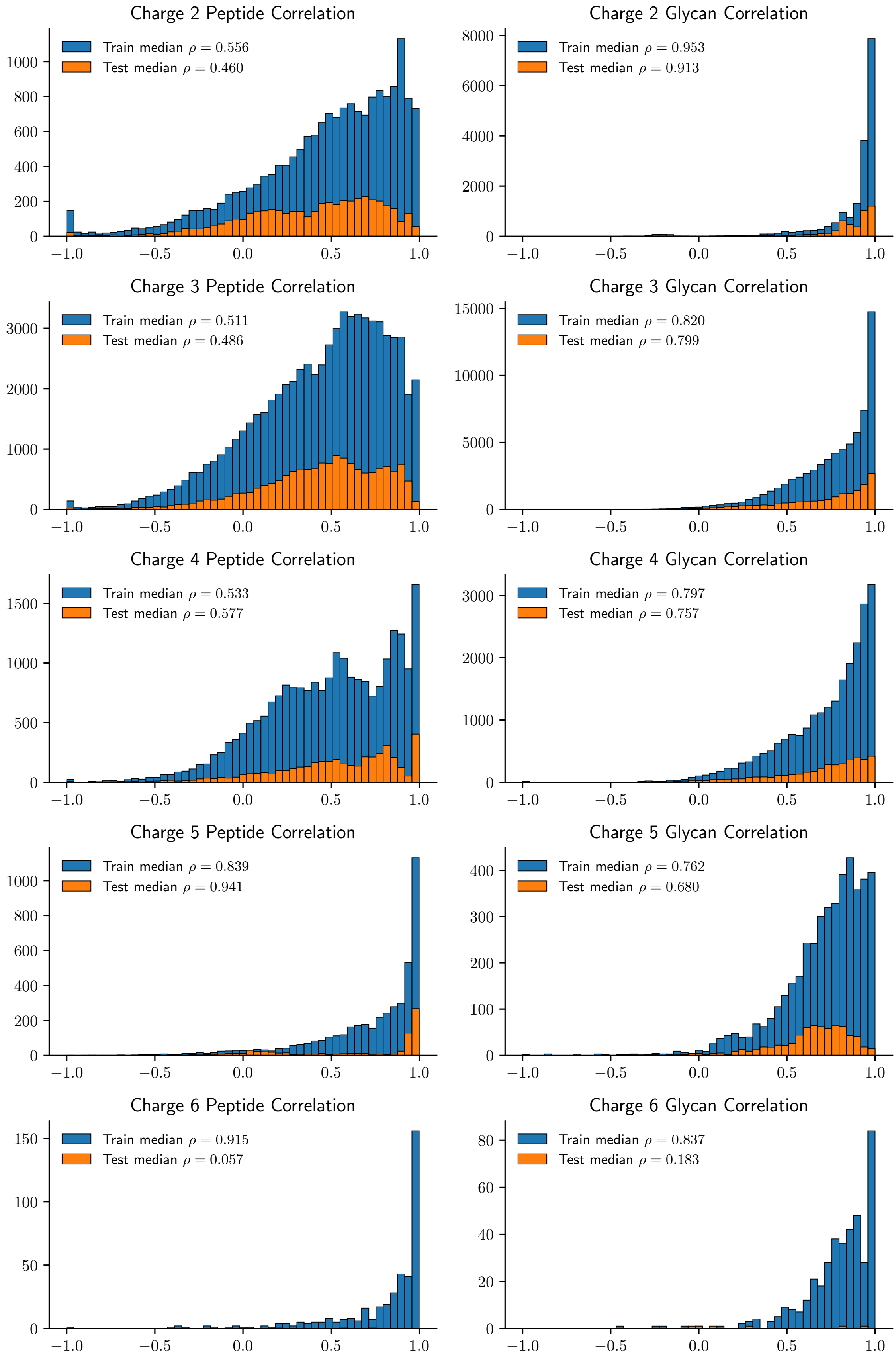

Figure 6: Spectrum intensity prediction correlation by charge state. The correlation between predicted and observed intensities summarized per charge state for the mouse tissue dataset

1.5 Fragmentation Model Features

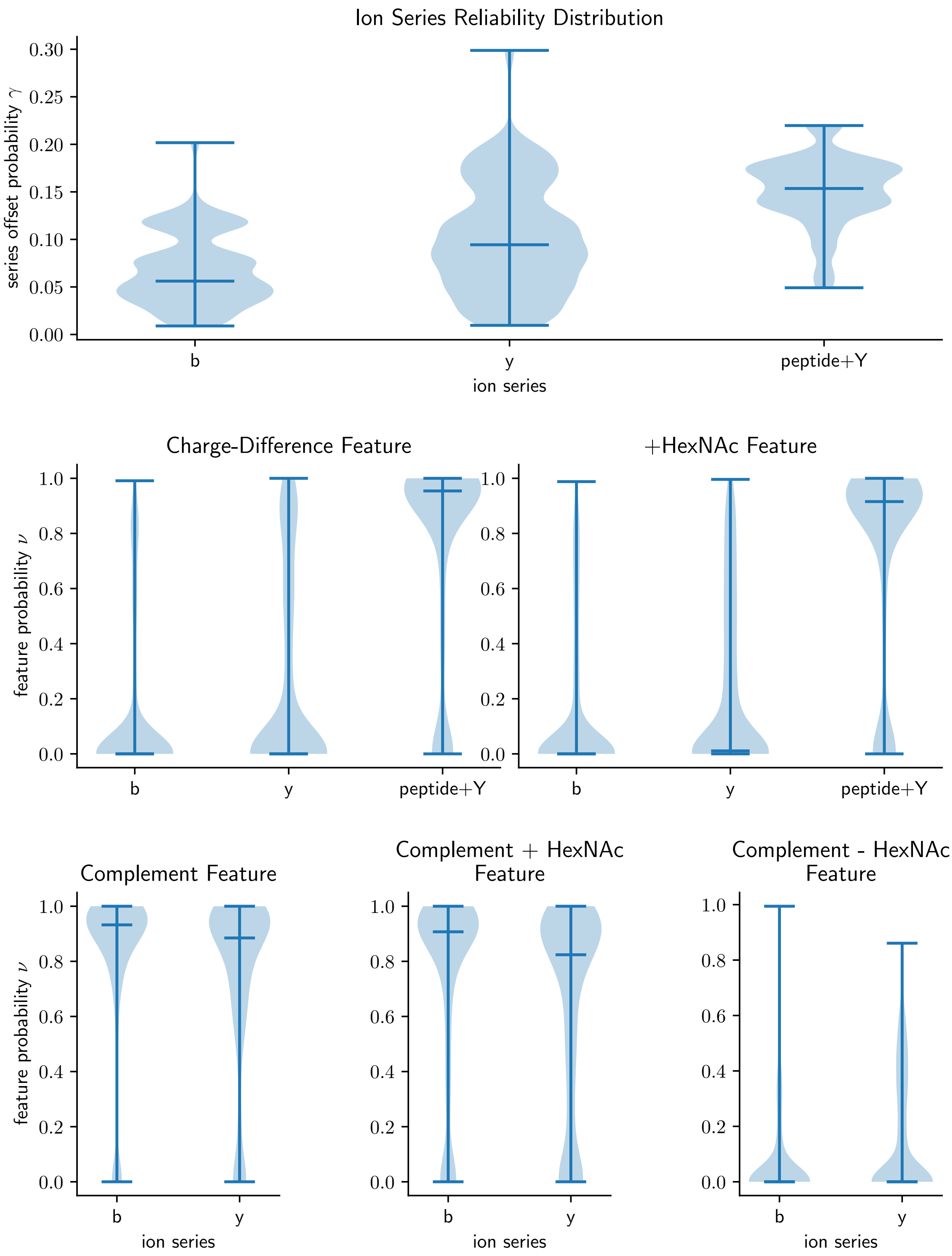

Figure 7: Fragmentation reliability model parameters. The probabilities associated with the ion series models and the associated common feature functions

1.6 Overlap with pGlyco3

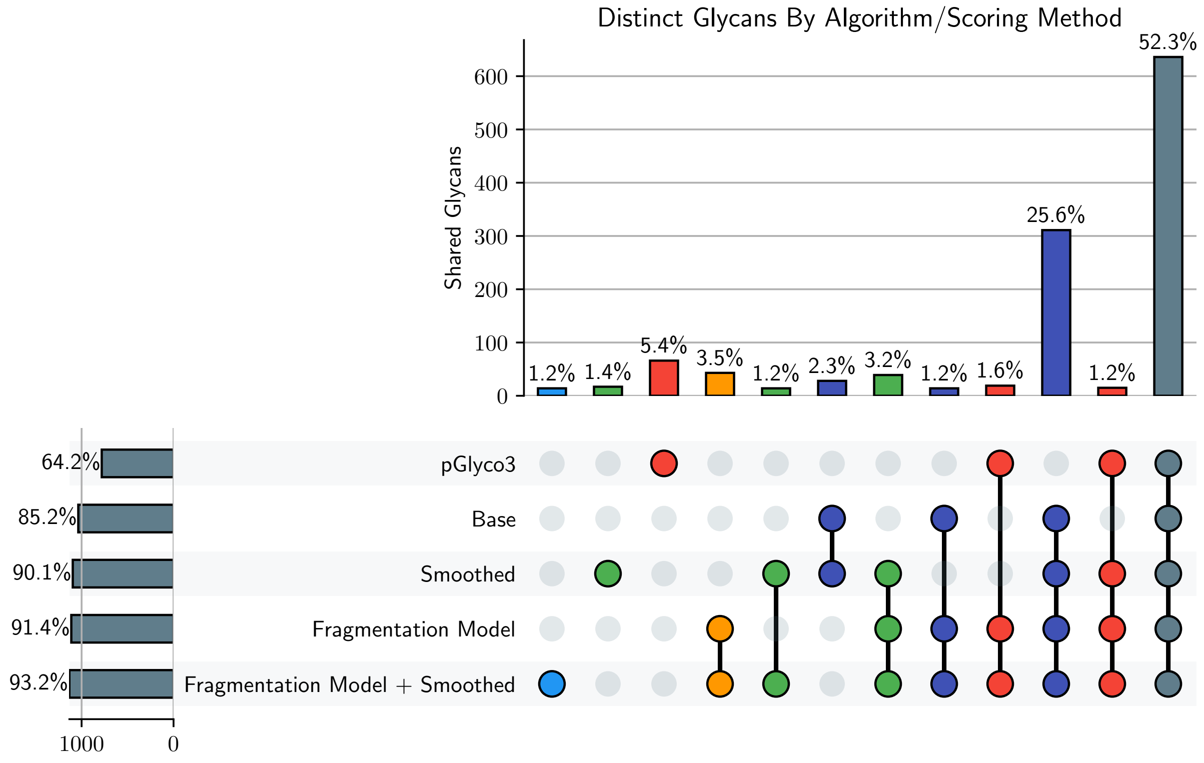

Figure 8: Glycans identified by strategy. The overlap in distinct glycan compositions identified by each tool or search strategy on the mouse tissue datasets. GlycReSoft identifies substantially more distinct glycan compositions than pGlyco3 due to its larger glycan database.

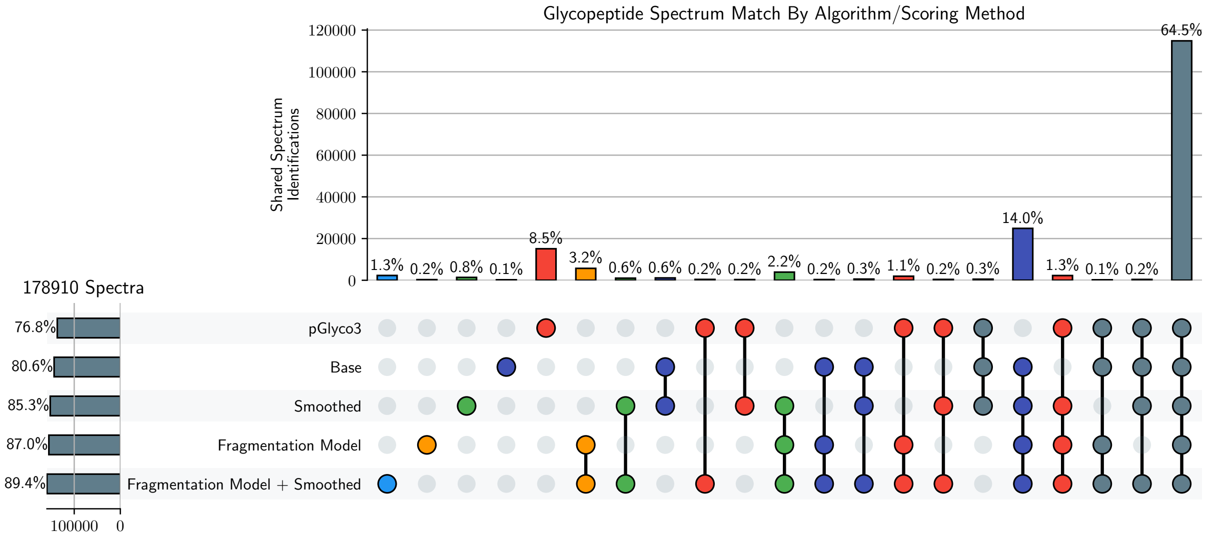

Figure 9: GPSMs identifications by strategy. The overlap in distinct GPSMs identified by each tool or search strategy. There are non-trivial populations of analytes that GlycReSoft missed that pGlyco3 was able to retain. We attribute this to both differences in how glycan composition coverage is calculated, and to observed differences in signal processing. While adduction state may play a role here, it is difficult to disentangle because of how GlycReSoft tracks ambiguous identifications until chromatographic feature resolution.

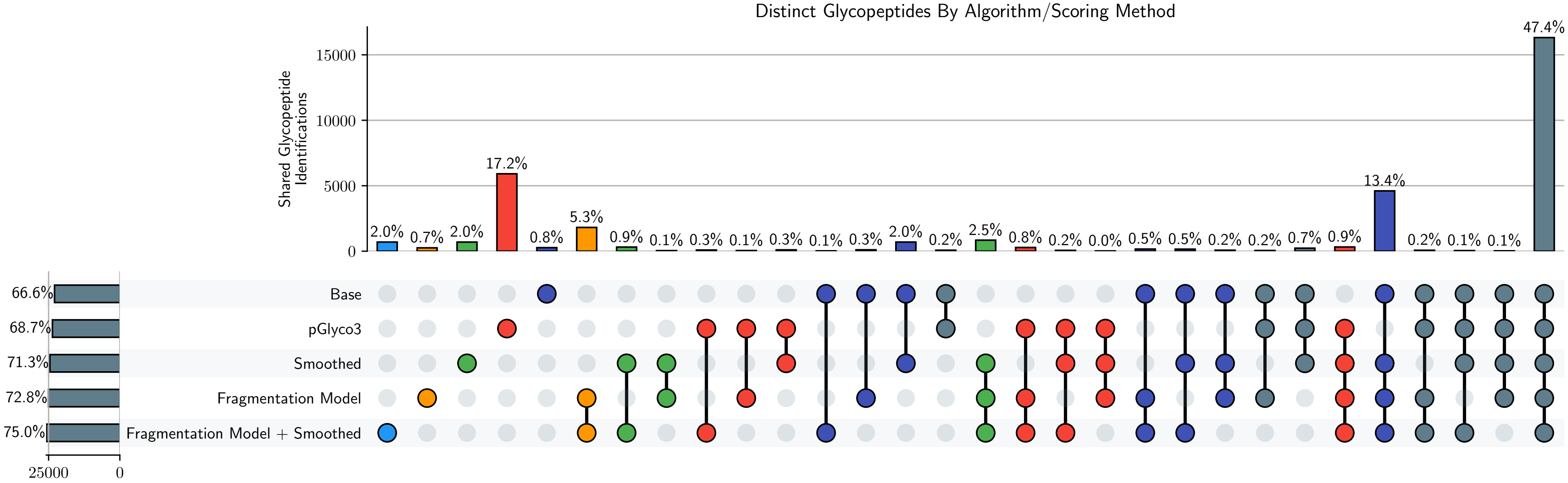

Figure 10: Glycopeptides identified by strategy. The overlap in distinct glycopeptides identified by each tool or search strategy. Unlike in Fig. 8, pGlyco3 shows a greater diversity in unique glycopeptides, despite identifying fewer spectra overall. This is due to GlycReSoft's retention time model consolidating glycoforms that would otherwise be randomly spread across overlapping adduction states of an analyte.

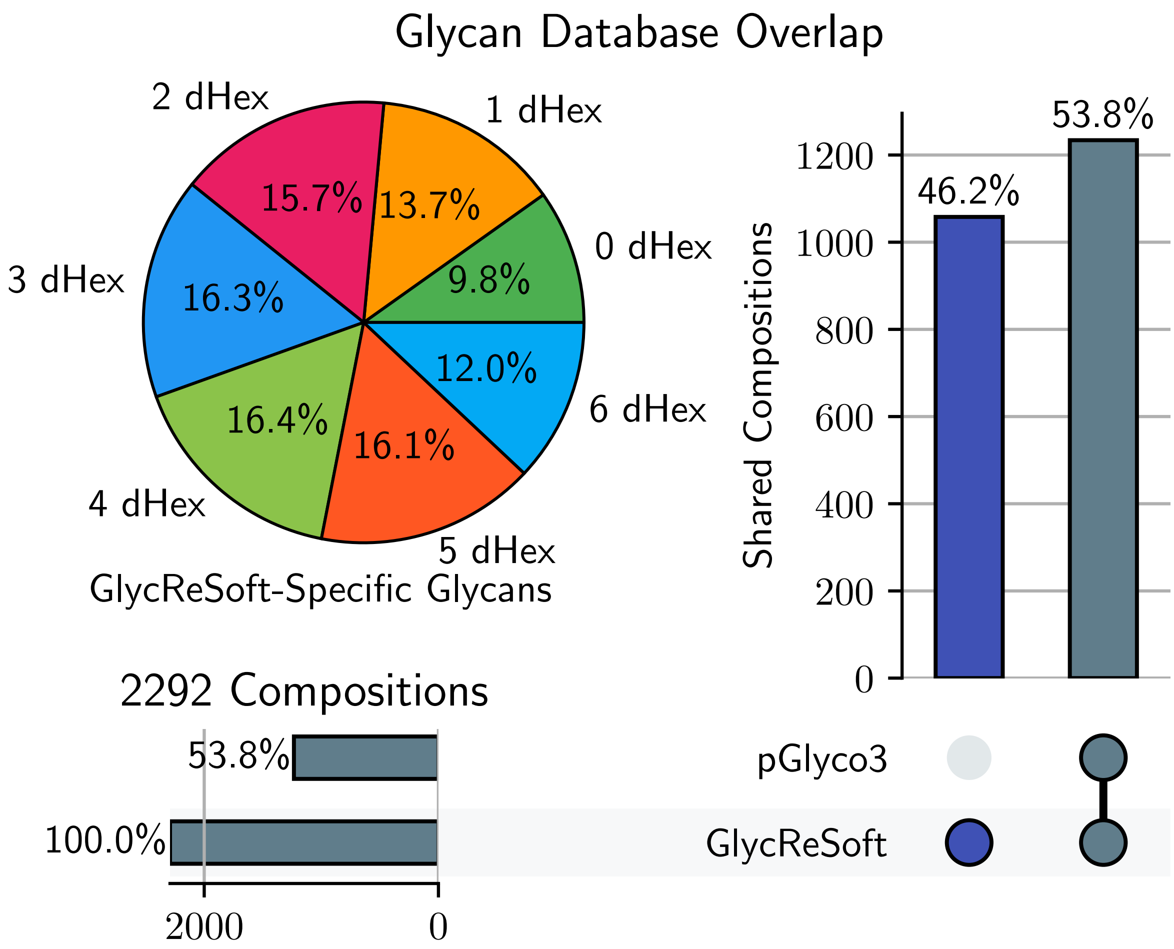

Figure 11: Glycan database overlap. The overlap between glycan databases used for the mouse tissue datasets shows that the GlycReSoft glycan database is nearly twice the size of the glycan database used by pGlyco3 after reducing its glycan structures to unique compositions. Most of the unique glycans are multiply fucosylated which is expected.

1.7 Example of site-specific glycome network smoothing

An expansion of the rescued glycoform shown in Figure 2j is shown in Figure 12. Here, we highlight several glycoforms that were missed under the base search algorithm but were supported by network smoothing at 1% FDR. In addition to good MS2 evidence, these examples are supported by agreement with the retention time model learned in the same sample with only the base search algorithm. While some of these identifications were present in the other replicates, a match-between-runs-based label propagation strategy based upon only precursor mass and retention time alignment has its own drawbacks which makes fully utilizing all viable MS2 spectra preferable.

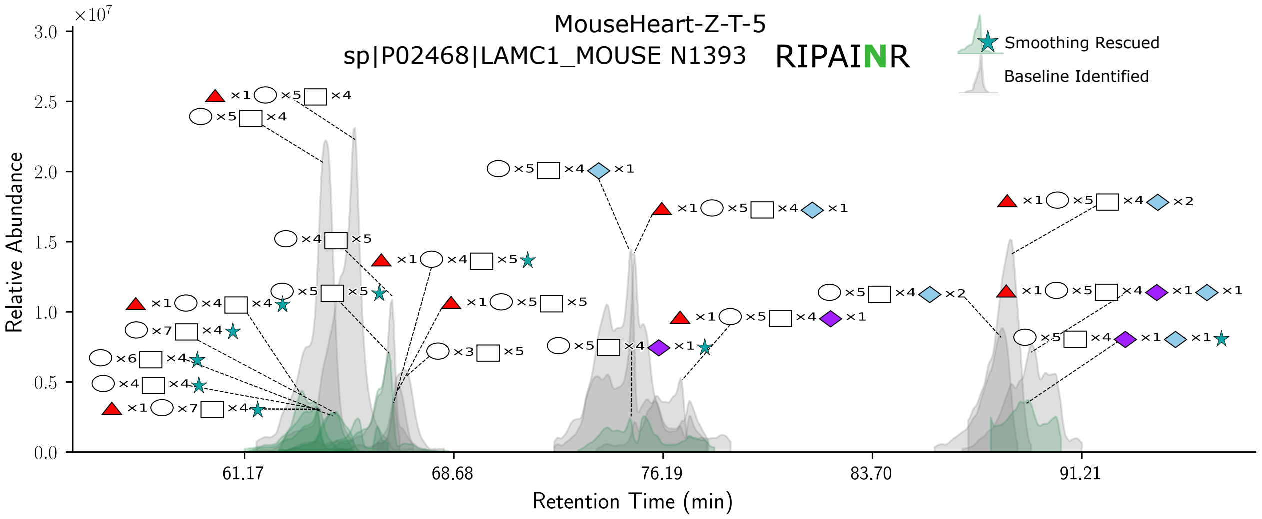

Figure 12: Example of a glycopeptide rescued by network smoothing in the context of its related glycoforms. The XICs for glycoforms of Laminin subunit gamma-1 (UniProt:P02468) in MouseHeart-Z-T-5 attached to the peptide sequence `RIPAINR` spanning N 1393. The XICs for glycoforms identified in the base strategy are shown in grey and those rescued by network smoothing are shown in green.

1.8 Yeast Entrapment Study

GlycReSoft has nearly twice the entrapment FDR of pGlyco3 based upon the the results shown in Figure 3b. GlycReSoft reports 40% more yeast peptides with non-yeast glycans, and 14x more mouse peptides in general at 1% FDR, compared to pGlyco3. The trapping glycans are shown in Table 1. The majority of these glycoforms are identified in an adducted state, and carry a deoxy-hexose. Additionally, GlycReSoft also identifies over 10 times as many mouse peptides with valid yeast glycans, with 70% of these coming from just the top three peptide backbones shown in Table 2. Two examples are shown in Figure 13, which highlight recurring properties in their set of 20 GPSMs each. All of the matches to GIAQNLTTEDLR have high abundance peaks that are unexplained and that differ from each other by monosaccharide units which suggests there are either multiple isolated precursors or, more likely, the identification is incorrect. In the case of ERNITR the glycan is fully explained but the peptide backbone is supported by few low abundance peaks though this is not unexpected for a short peptide with only partial proton mobility. Some of these peptide backbones do appear in the unfiltered pGlyco3 output, but any number of factors between small differences in scoring procedures, gain/loss of product ion peaks, and the way that targets and decoys are filtered post-search may be the culprit. For example, scan 10909 from "cwq_mix2-2_726.raw" matches to a decoy peptide in pGlyco3's results and does not appear in the final output, but does in GlycReSoft's as a match to ERNITR{Hex:9; HexNAc:2}, although it narrowly passes the 1% FDR threshold.

glycan composition	mass shift	count	in pGlyco3 database
{Fuc:1; Hex:8; HexNAc:2}	Ammonium	22	True
{Fuc:1; Hex:8; HexNAc:2}	Ammonium * 2	14	True
{Fuc:1; Hex:7; HexNAc:2}	Ammonium	12	True
{Fuc:1; Hex:9; HexNAc:2}	Ammonium * 2	9	True
{Fuc:3; Hex:10; HexNAc:7}	Ammonium	7	True
{Fuc:1; Hex:9; HexNAc:2}	Ammonium	7	True
{Fuc:1; Hex:7; HexNAc:2}	Ammonium * 2	5	True
{Hex:5; HexNAc:4; Neu5Ac:2}	Unmodified	4	True
{Fuc:3; Hex:6; HexNAc:4}	Unmodified	4	True
{Hex:6; HexNAc:4; Neu5Ac:2}	Unmodified	4	True
{Fuc:2; Hex:11; HexNAc:8}	Ammonium	2	False
{Fuc:1; Hex:11; HexNAc:5}	Unmodified	2	False
{Hex:7; HexNAc:6}	Ammonium	2	True
{Fuc:4; Hex:4; HexNAc:4}	Unmodified	2	True
{Fuc:3; Hex:4; HexNAc:4; Neu5Gc:1}	Unmodified	2	True
{Fuc:3; Hex:4; HexNAc:4}	Unmodified	2	True
{Fuc:5; Hex:12; HexNAc:7}	Ammonium * 2	1	False
{Fuc:1; Hex:7; HexNAc:3}	Unmodified	1	True
{Hex:7; HexNAc:6}	Unmodified	1	True
{Hex:9; HexNAc:6}	Ammonium	1	True
{Hex:5; HexNAc:6}	Unmodified	1	True
{Hex:6; HexNAc:9}	Ammonium * 2	1	True
{Fuc:6; Hex:11; HexNAc:8}	Unmodified	1	False
{Fuc:3; Hex:7; HexNAc:4}	Unmodified	1	True
{Fuc:3; Hex:5; HexNAc:4}	Unmodified	1	True
{Fuc:1; Hex:7; HexNAc:2}	Unmodified	1	True
{Hex:6; HexNAc:6}	Unmodified	1	True
{Hex:8; HexNAc:6}	Ammonium	1	True
{Fuc:2; Hex:8; HexNAc:7}	Ammonium * 2	1	True
{Fuc:3; Hex:6; HexNAc:7}	Ammonium * 2	1	True
{Fuc:5; Hex:5; HexNAc:4}	Unmodified	1	True
{Hex:5; HexNAc:5}	Ammonium * 2	1	True
{Hex:7; HexNAc:10}	Unmodified	1	True
{Fuc:3; Hex:3; HexNAc:4}	Unmodified	1	True
{Fuc:4; Hex:5; HexNAc:4}	Unmodified	1	True
{Hex:9; HexNAc:6}	Unmodified	1	True
{Hex:6; HexNAc:10}	Ammonium * 2	1	True
{Hex:6; HexNAc:6}	Ammonium	1	True

Table 1: Non-yeast glycan compositions GlycReSoft identifies in entrapment dataset. The non-yeast glycan compositions that contributed to GlycReSoft's entrapment FDR. The major contributors are high mannose-like compositions with a dHex, and are almost universally ammonium adducted.

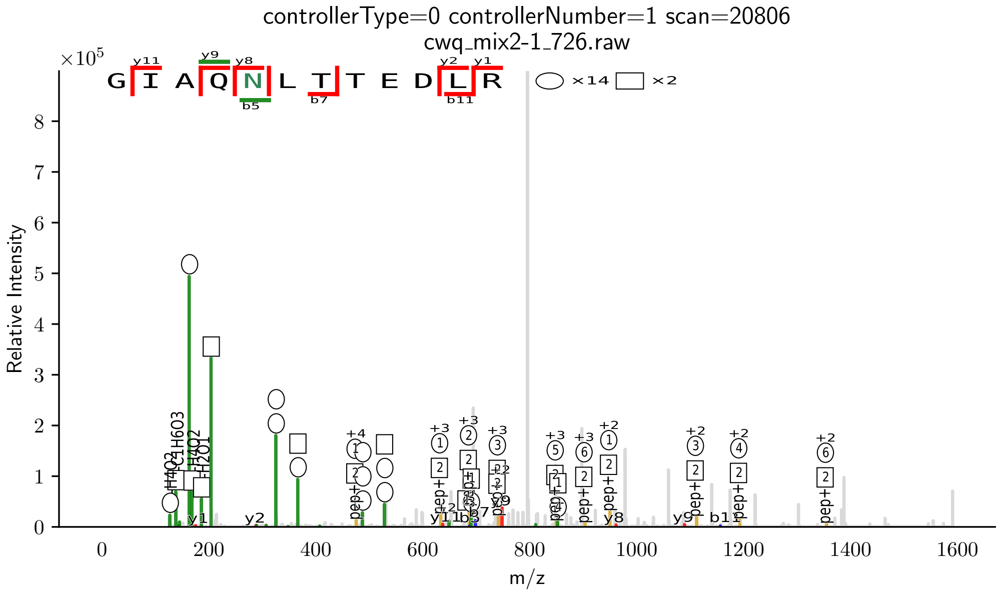

(a)

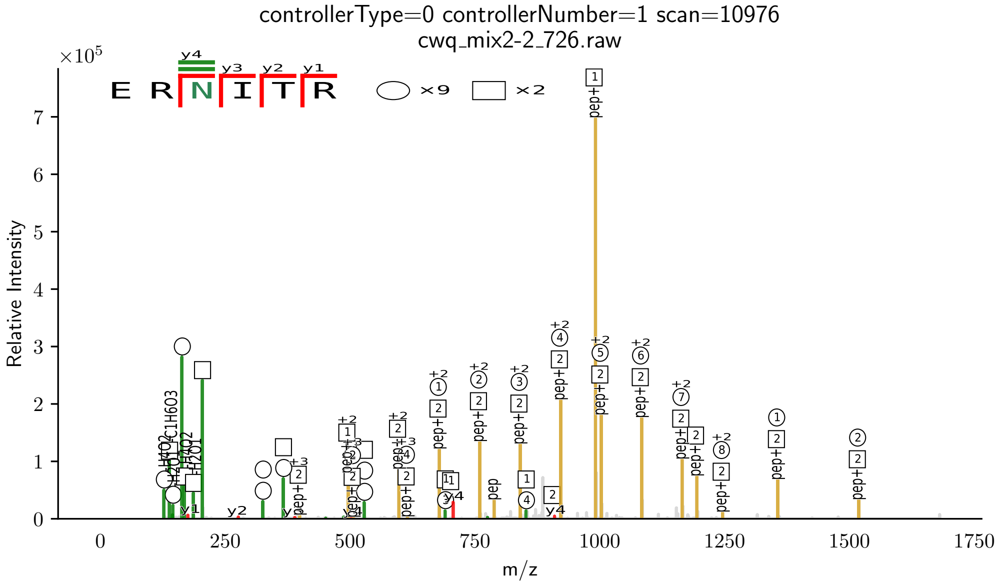

(b)

Figure 13: Annotated spectra for primary drivers of entrapment FDR. (a) A GPSM for the mouse peptide GIAQNLTTEDLR from UniProt:Q8R0S9, there are several high intensity peaks that have gone unassigned and appear to have monosaccharide unit spacing,(b) A GPSM for the mouse peptide ERNITR from UniProt:Q8VDQ1, while the glycan is very well explained, the short peptide backbone is defined by only four low abundance peaks.

peptide backbone	count	distinct glycoforms
K.GIAQNLTTEDLR.S	17	2
R.INSTDR.L	11	2
K.NVTEKK.L	5	1
N.EVPAQN.L	2	2
K.ERNITR.E	2	1
K.LEALMASNDSANR.T	2	2
K.NLCMSTYKR.F	2	1
L.ENLSFSCPK.D	1	1
K.AALEDSNGSSELQEIMR.R	1	1
R.NMTGYK.T	1	1
R.MVENSSPR.A	1	1
R.TNITQEHFSHR.E	1	1
K.YTAVQRNCSDR.E	1	1
R.NATTGR.I	1	1
K.NYTVSETSTTK.S	1	1
D.TPANCTYP.D	1	1
K.KDVNLSK.T	1	1

Table 2: Mouse peptides GlycReSoft identifies in the yeast entrapment dataset. The mouse peptides that contributed to GlycReSoft's entrapment FDR. There are three major contributing peptide backbones that account for 64% of cases.

1.9 Intra-cluster Similarity Validation

To support the additional methods we introduce in this work, including modifications to the FDR estimation procedure, we did a post-search spectral similarity calculation for each identified glycopeptide in the mouse tissue dataset. Within each MS run, we clustered GPSM from the same glycopeptide in the same charge state and adduction state, and computed the average dot product spectral similarity between all members of a cluster. The average similarities for all non-singleton clusters are shown in Table 3. Each additional modeling layer added more clusters without substantially altering the average similarities for each run.

Strategy	Mean Intra-Cluster Similarity	# of Clusters
Base	0.952	89403
Fragmentation Model	0.952	93266
Smoothed	0.952	93027
Fragmentation Model + Smoothed	0.951	94911

Table 3: Average intra-cluster similarities by identification strategy. The average spectrum similarity within each glycopeptide identification for each strategy for the mouse tissue datasets, with clusters of size 1 discarded. A cluster was required to match in glycopeptide, precursor charge, and adduction state.

1.10 Predicted Spectra

1.11 *N*-Glycopeptide Spectra

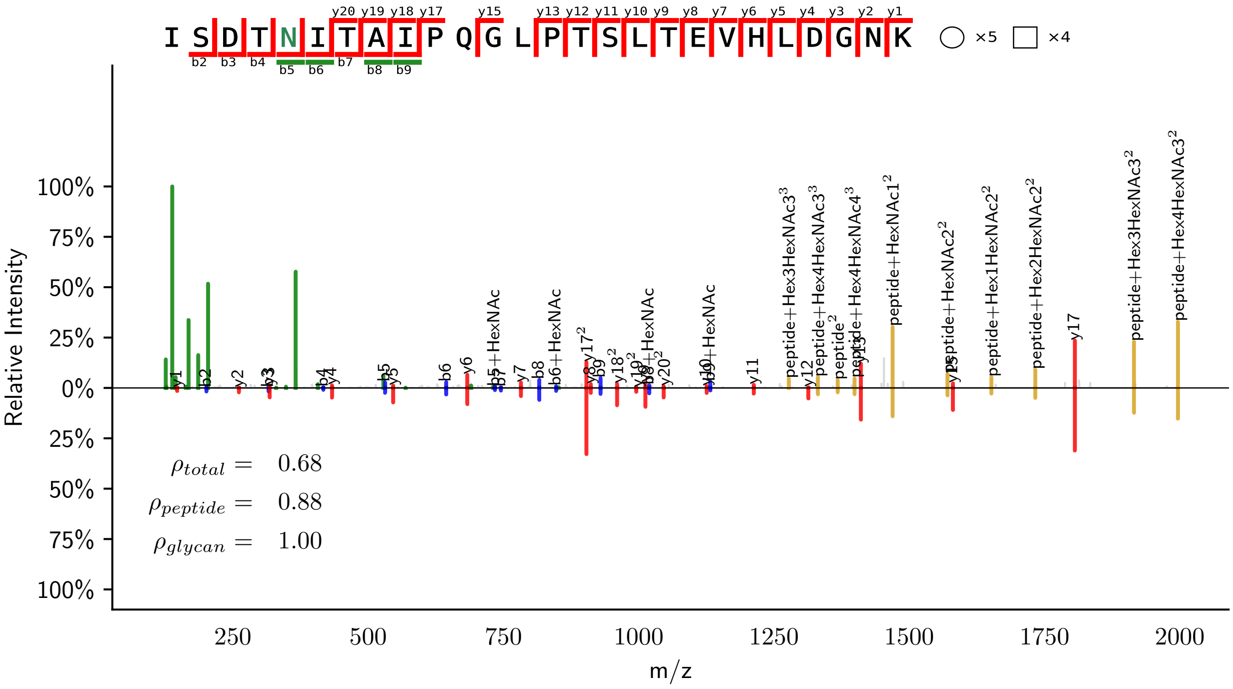

(a)

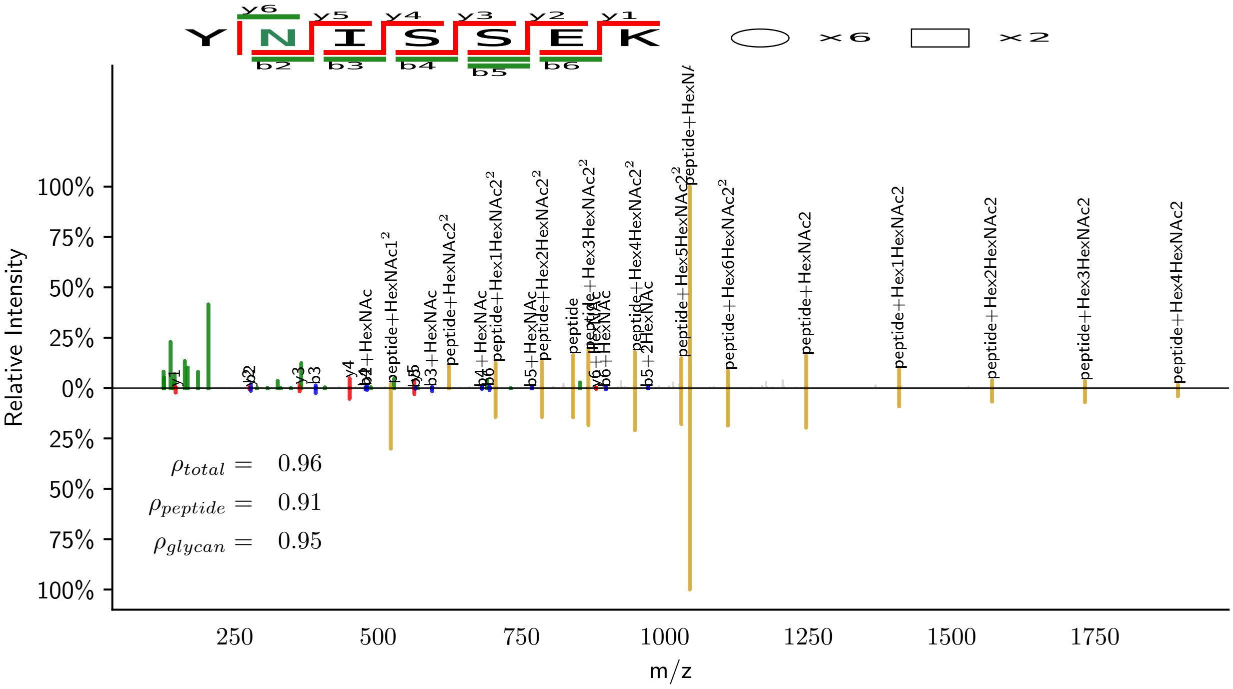

(b)

Figure 14: Mouse predicted spectra from the training set. Mouse Tissue predicted *N*-glycopeptide spectra examples

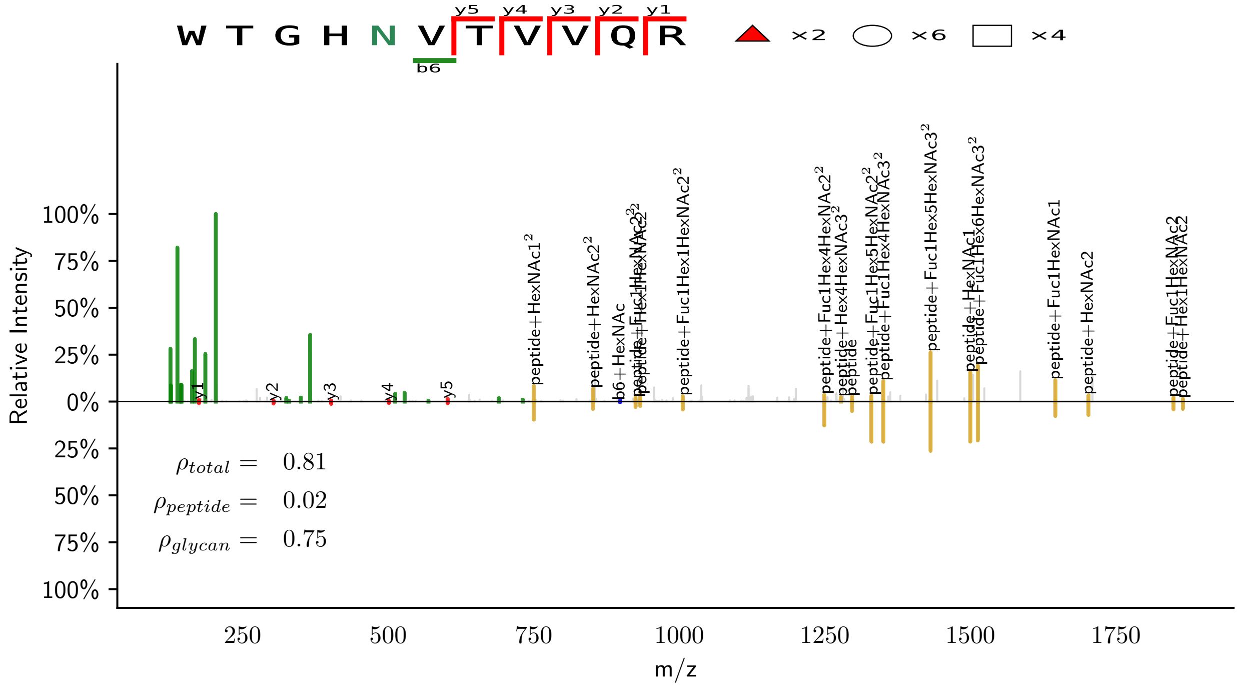

(a)

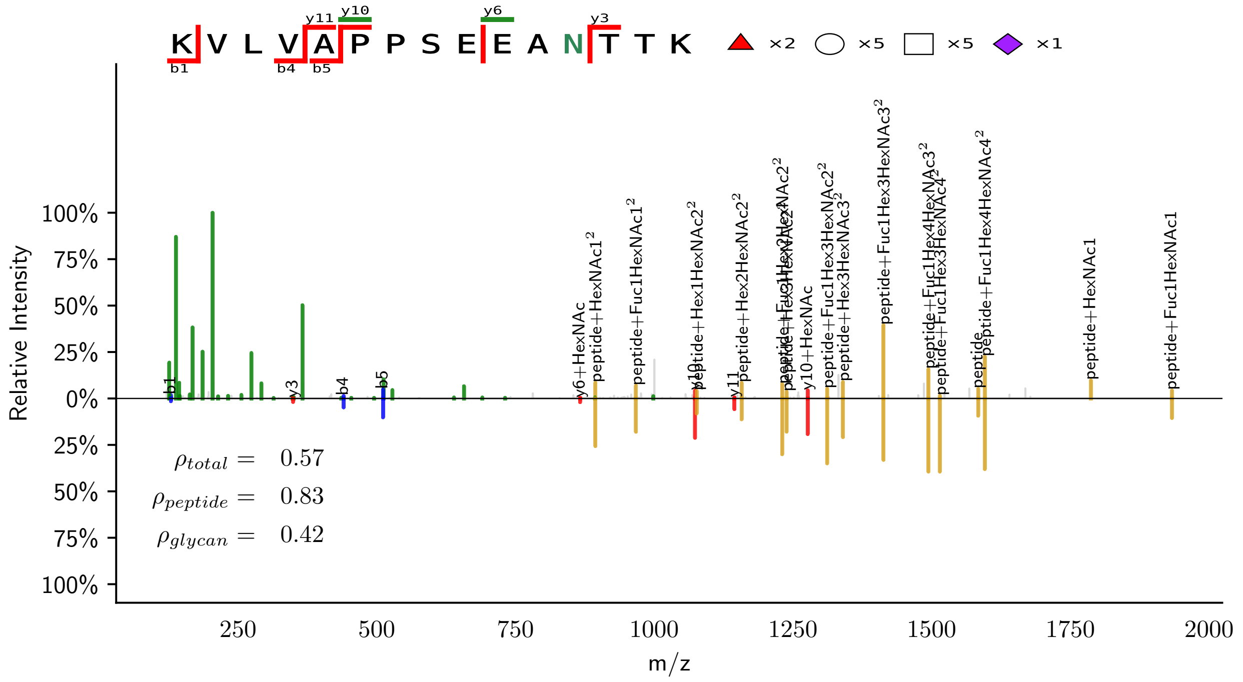

(b)

Figure 15: Mouse predicted spectra from the testing set. Mouse Brain predicted *N*-glycopeptide spectra examples

1.12 *O*-Glycopeptide Spectra

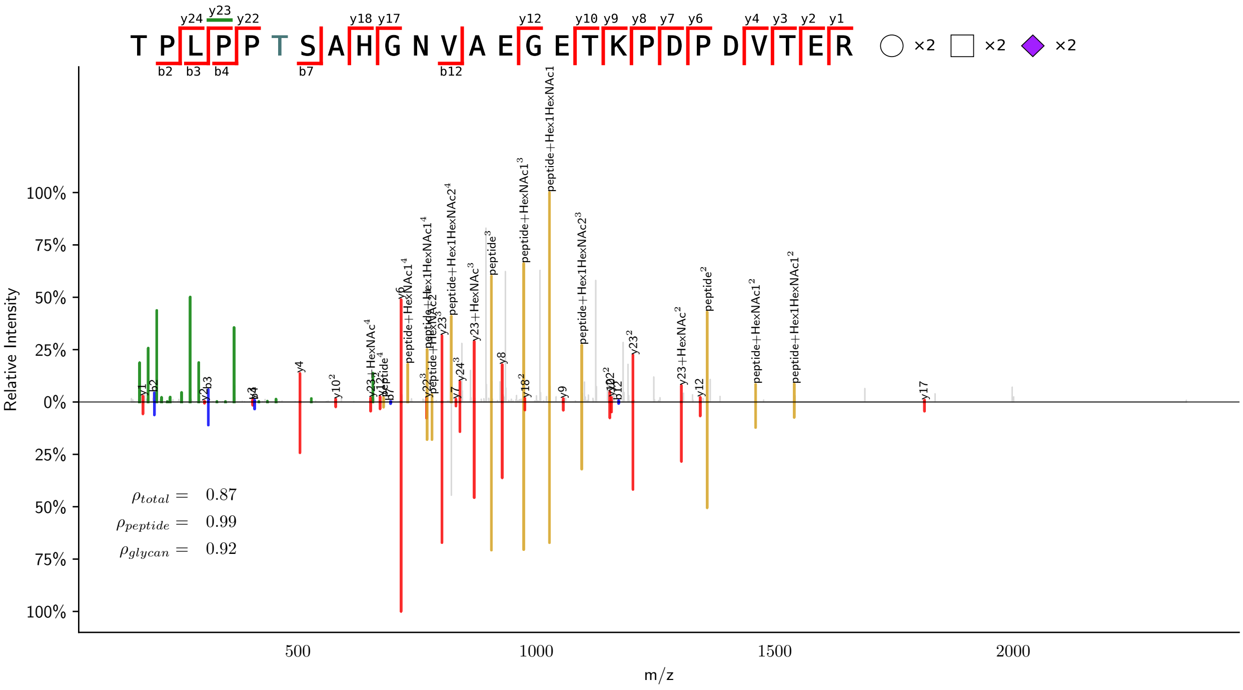

(a)

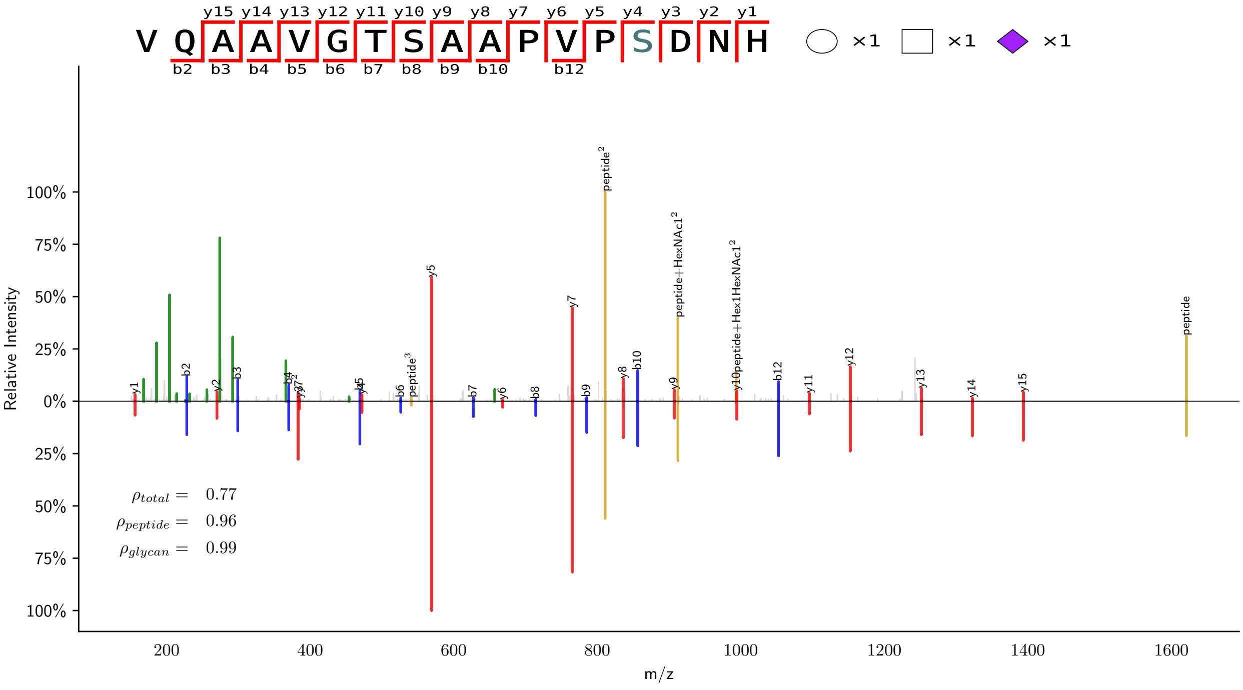

(b)

Figure 16: O-glycopeptide spectrum prediction. *O*-Glycopeptide spectral predictions appear strong, but they are likely over-fit due to the small amount of available training data for each partition

1.13 Fitted Retention Time Models

All mouse tissue datasets' retention time models are shown in this section in the following form:

- Top panel: Number of glycopeptides available at each time point for local model fitting.
- Middle panel: Monosaccharide coefficients with their approximate local confidence intervals computed from the average of L1-regularized weighted least squares models for that point in time.
- Bottom panel: Residual error of training observations over time, with horizontal stripes denoting the zero line, the 25th and 75th quantiles of the $\alpha = 0.01$ prediction interval widths (inner), and the uncertainty estimate padded prediction interval bounds. Coefficients are interpolated to reflect the weight that observation time distance to model centroid had.
- All plots for the same sample share an x-axis, with vertical stripes denoting time bin edges over which training observations are partitioned.

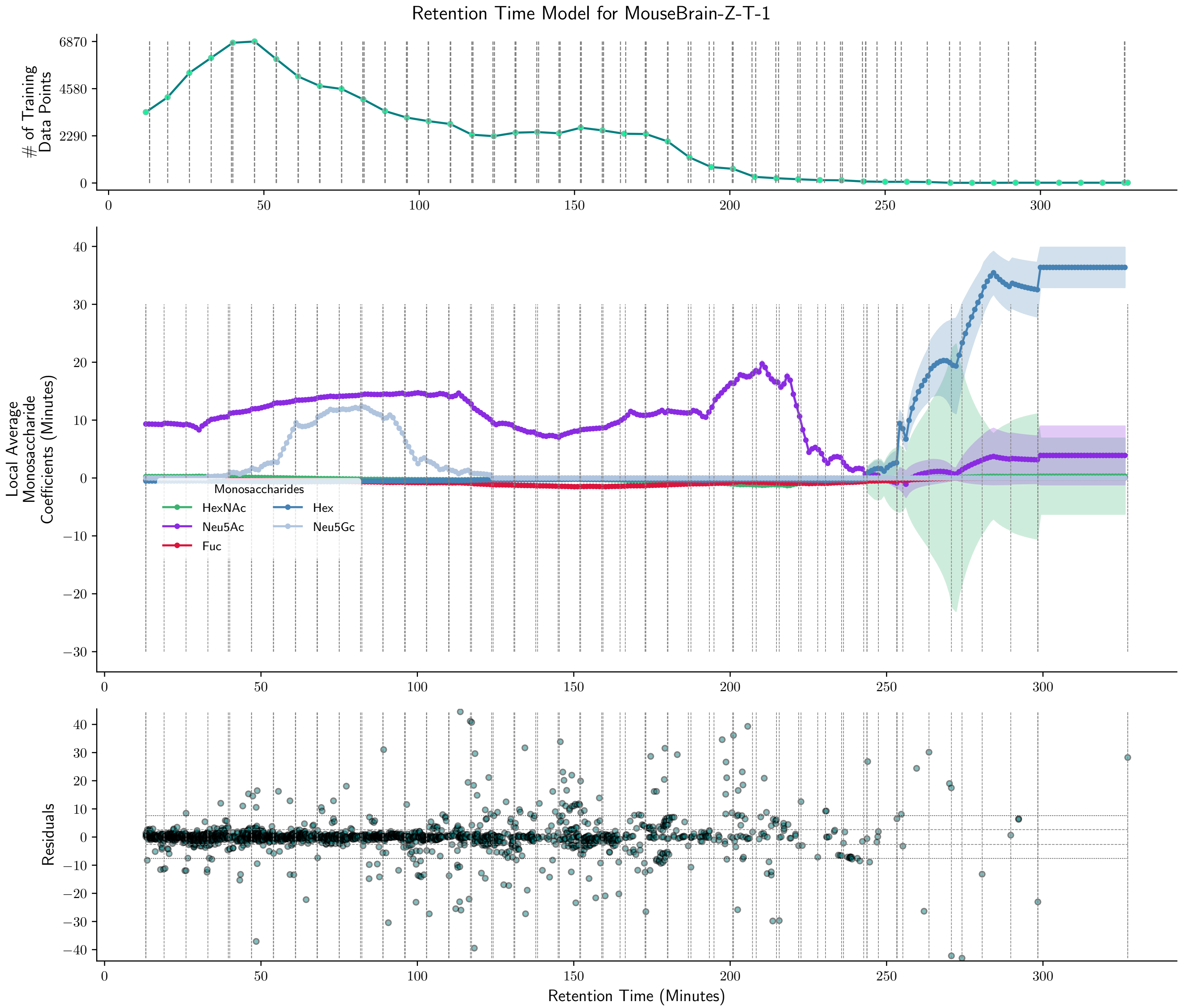

Figure 17: Extended retention time figure for Mouse Brain1. Extended retention time figure for Mouse Brain1

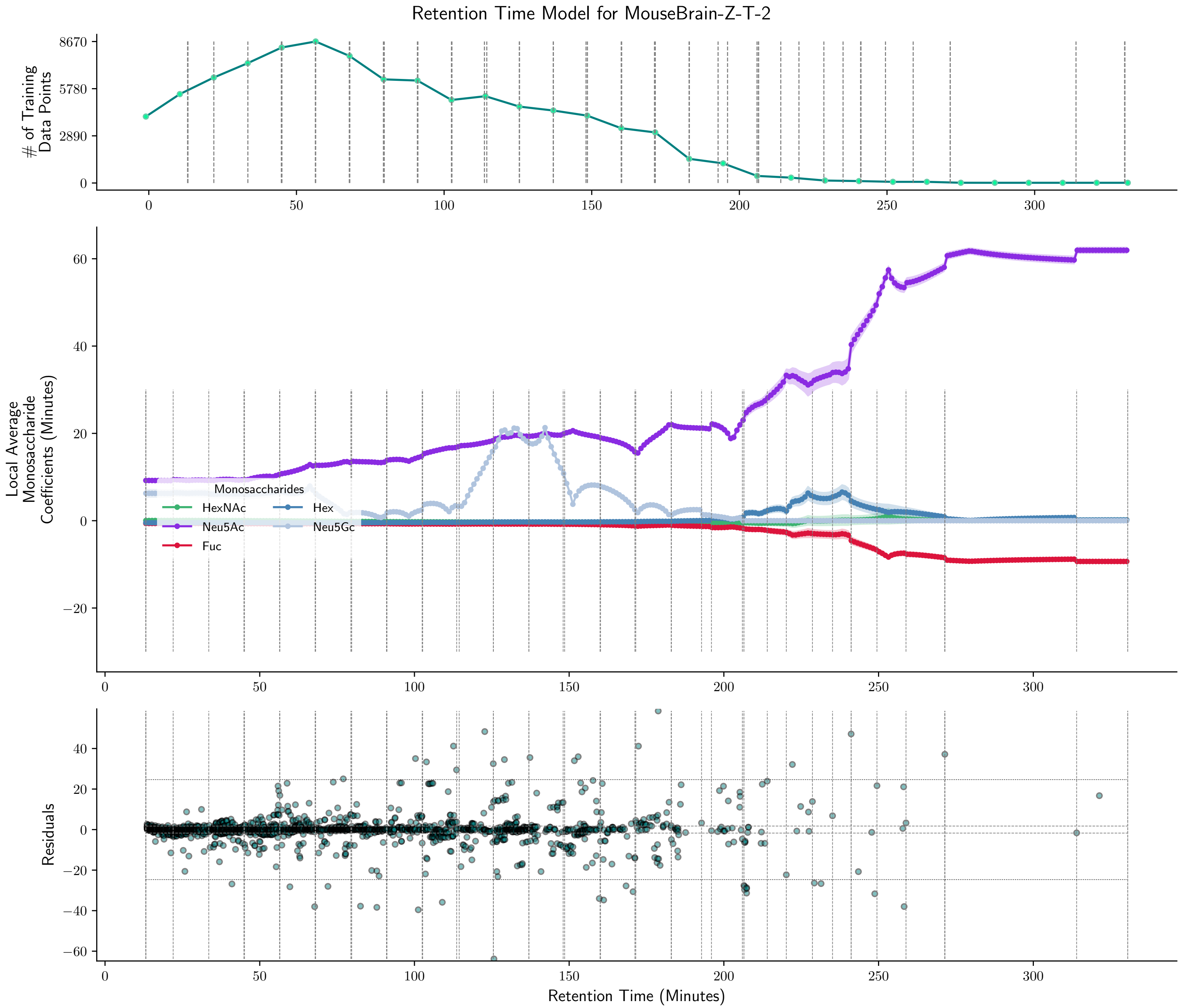

Figure 18: Extended retention time figure for Mouse Brain2. Extended retention time figure for Mouse Brain2

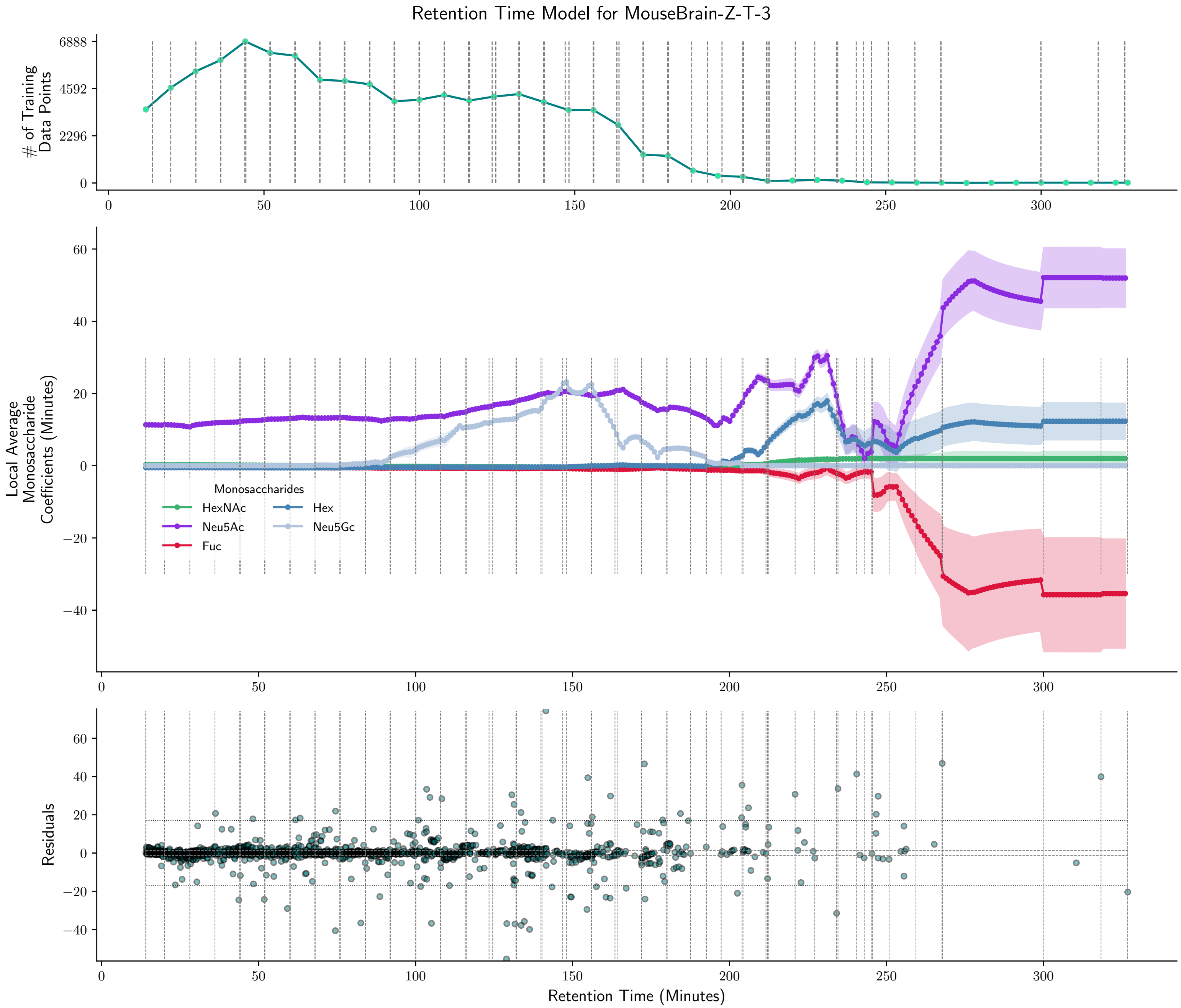

Figure 19: Extended retention time figure for Mouse Brain3. Extended retention time figure for Mouse Brain3

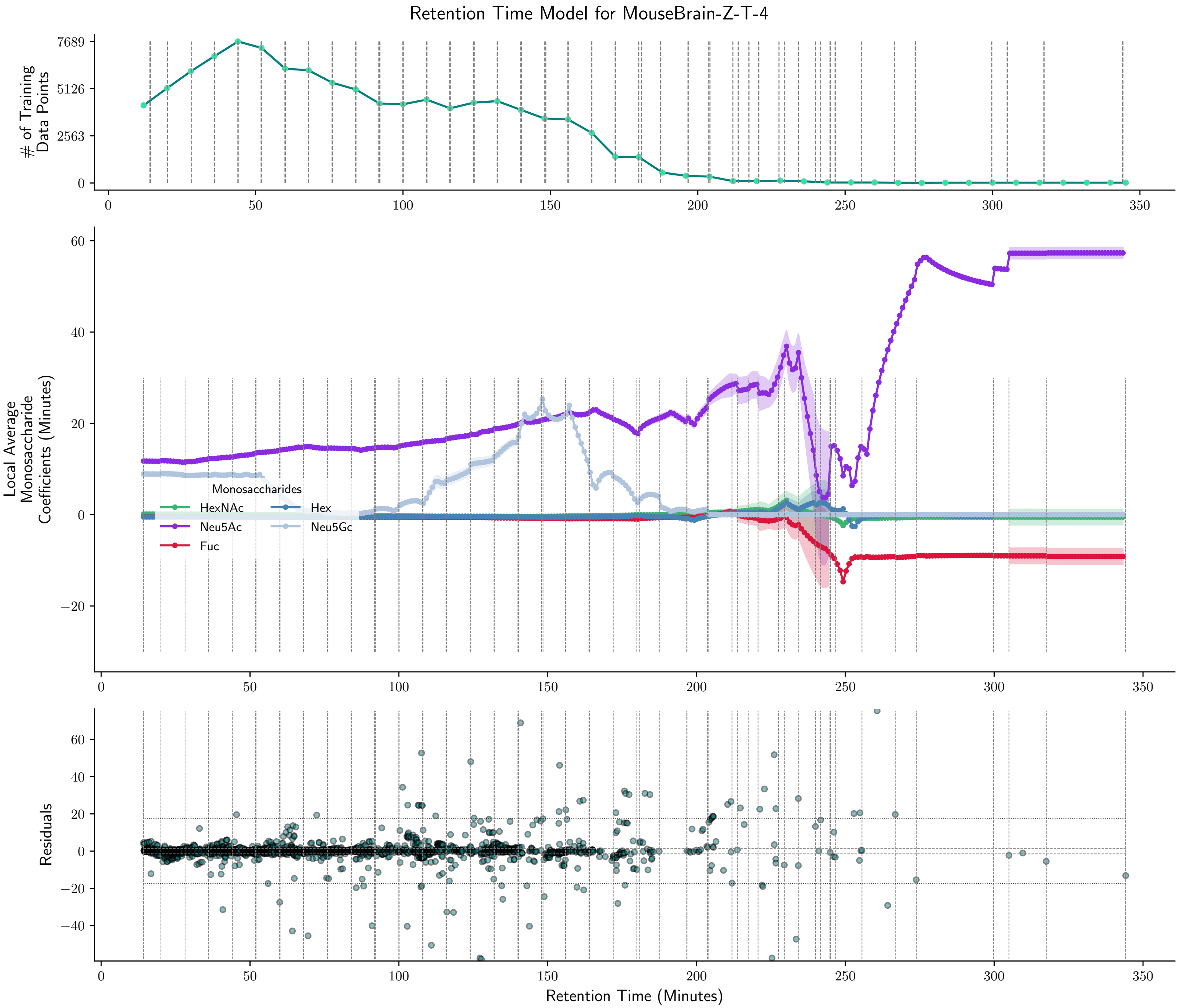

Figure 20: Extended retention time figure for Mouse Brain4. Extended retention time figure for Mouse Brain4

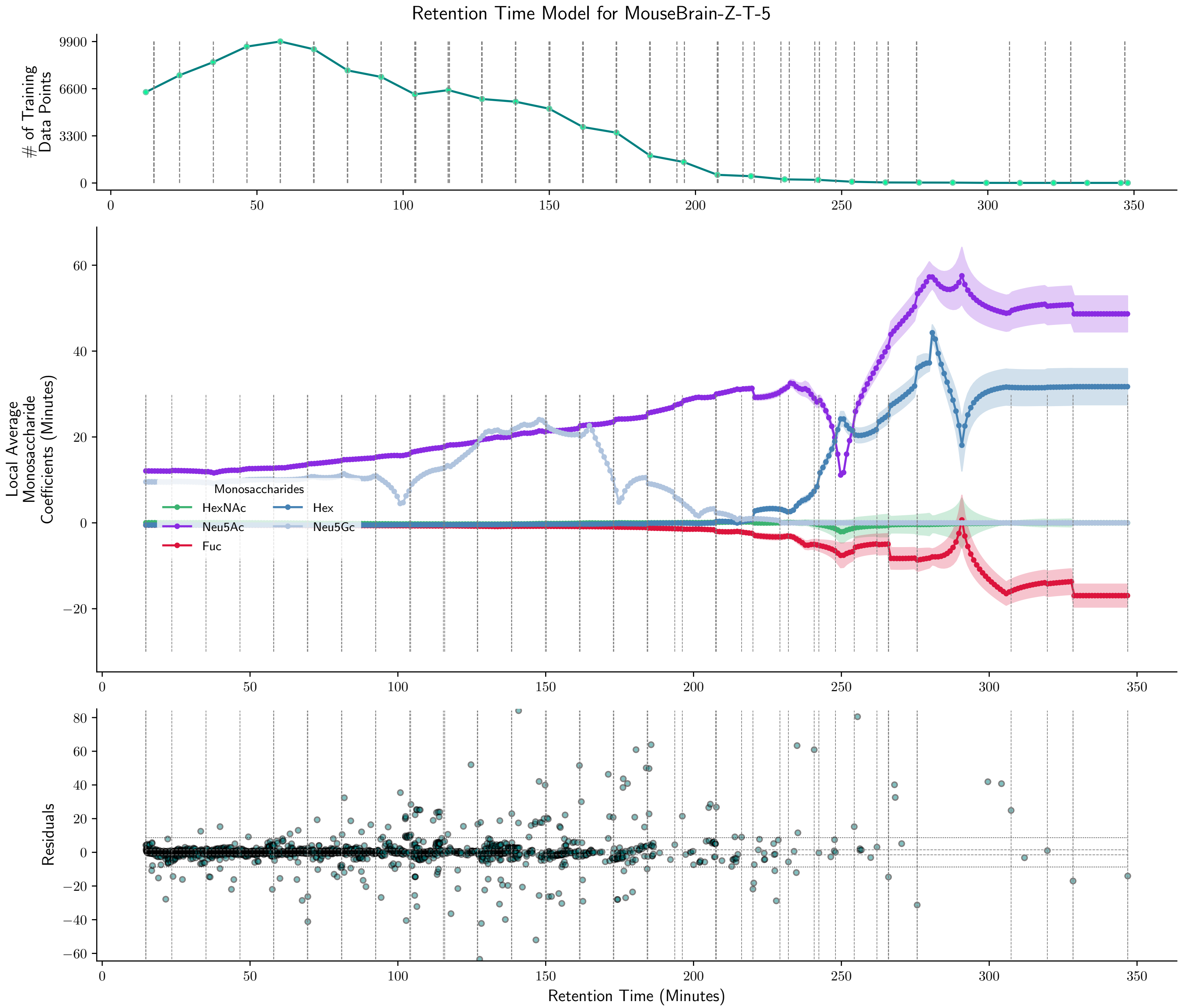

Figure 21: Extended retention time figure for Mouse Brain5. Extended retention time figure for Mouse Brain5

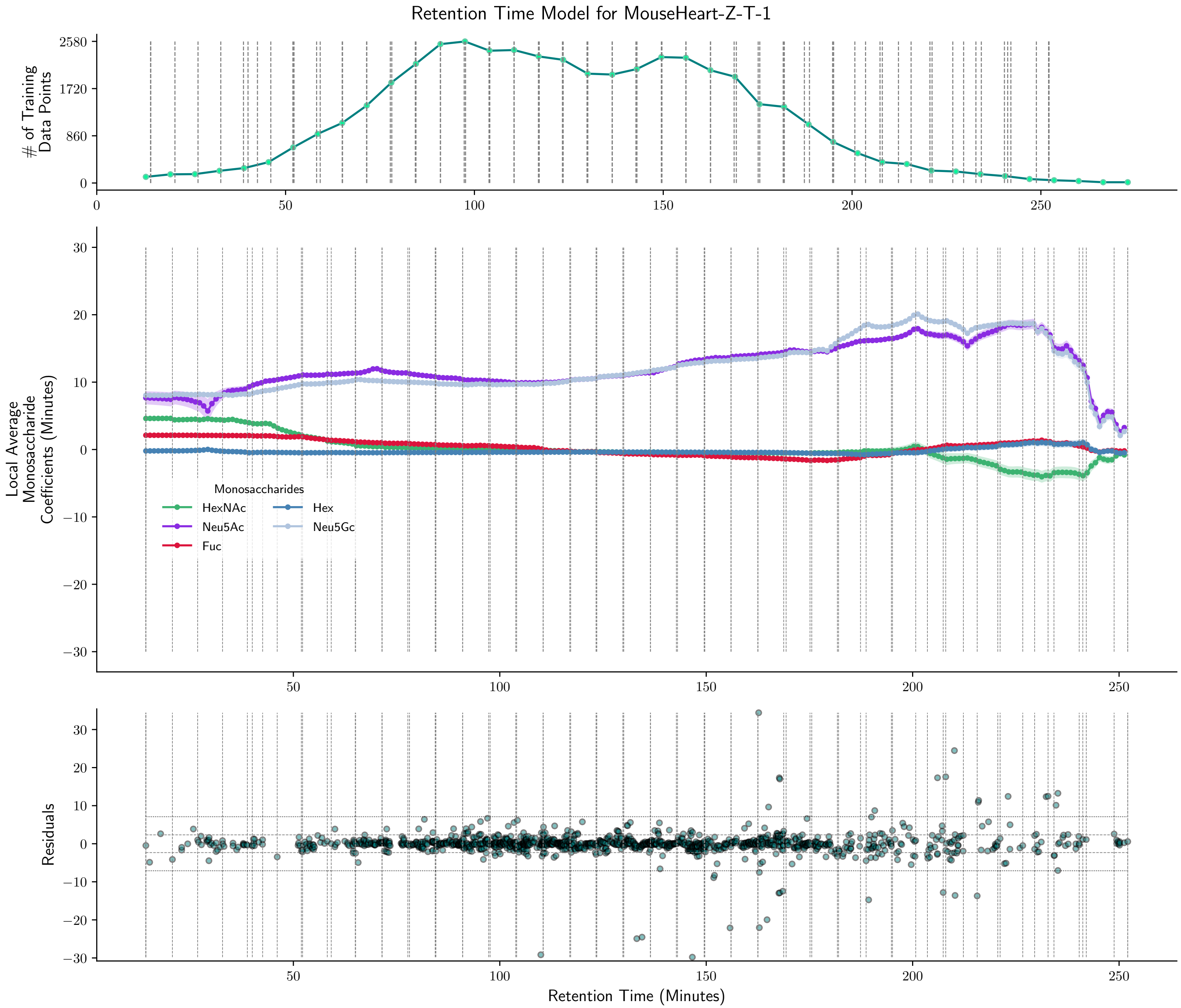

Figure 22: Extended retention time figure for Mouse Heart1. Extended retention time figure for Mouse Heart1

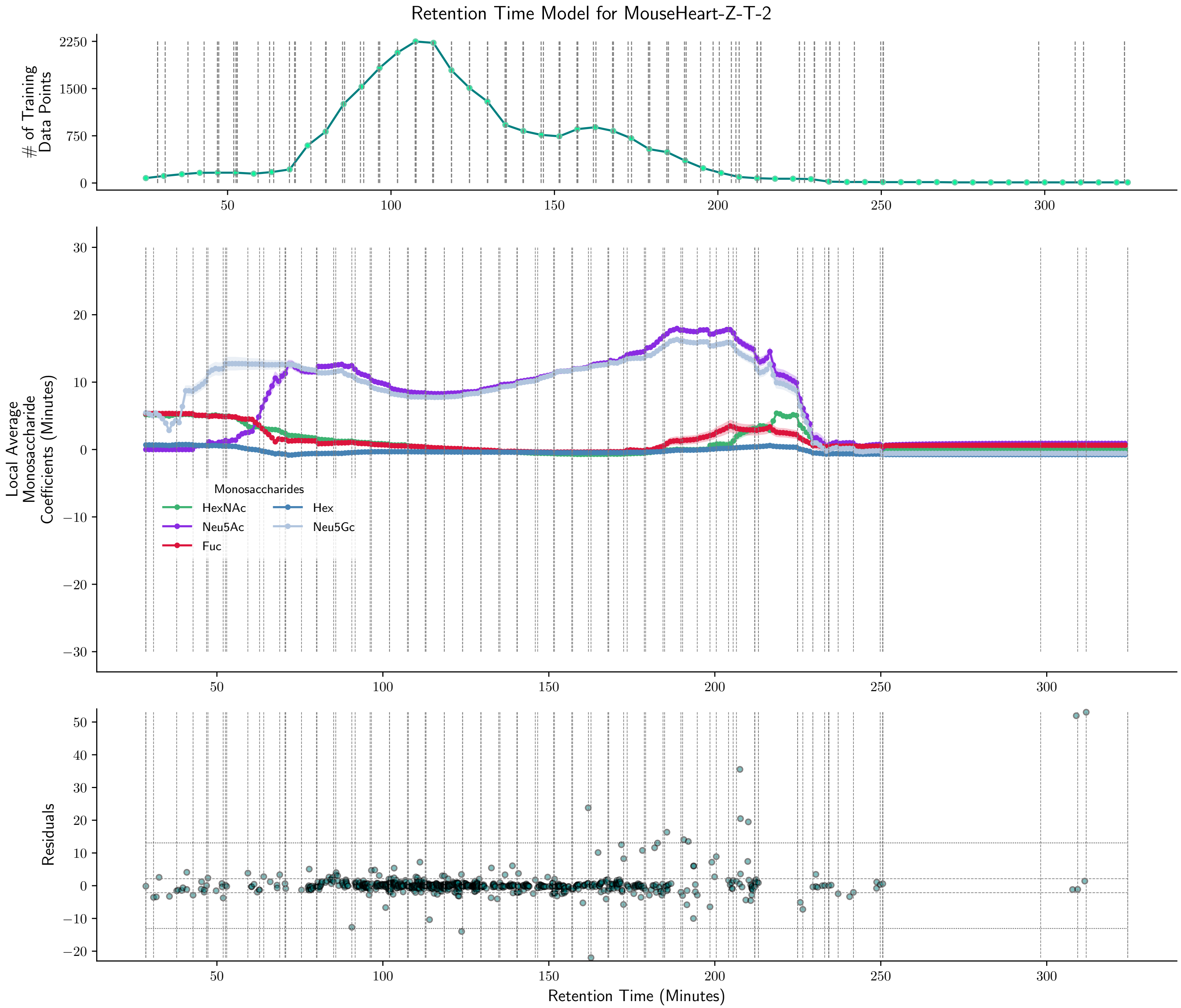

Figure 23: Extended retention time figure for Mouse Heart2. Extended retention time figure for Mouse Heart2

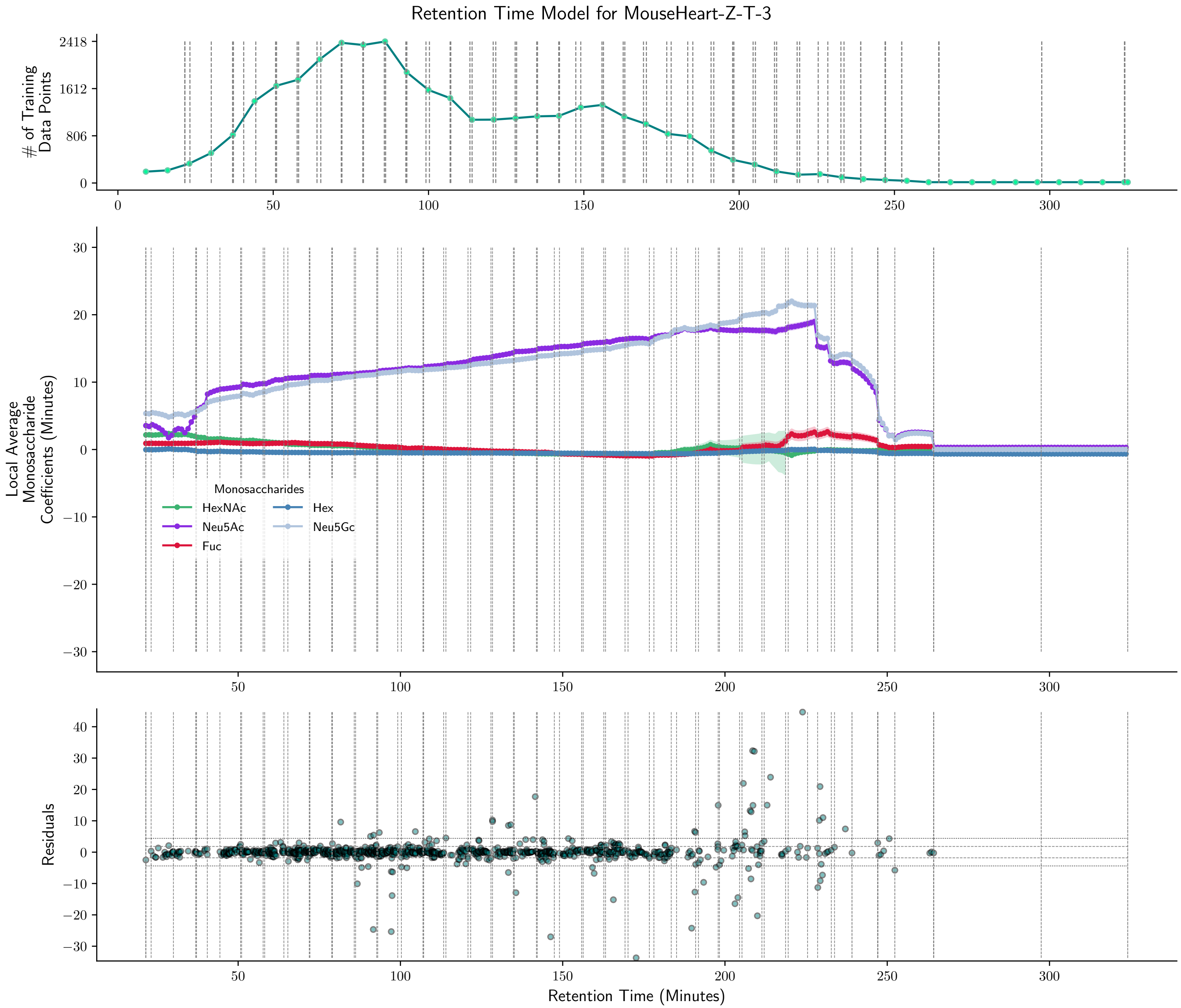

Figure 24: Extended retention time figure for Mouse Heart3. Extended retention time figure for Mouse Heart3

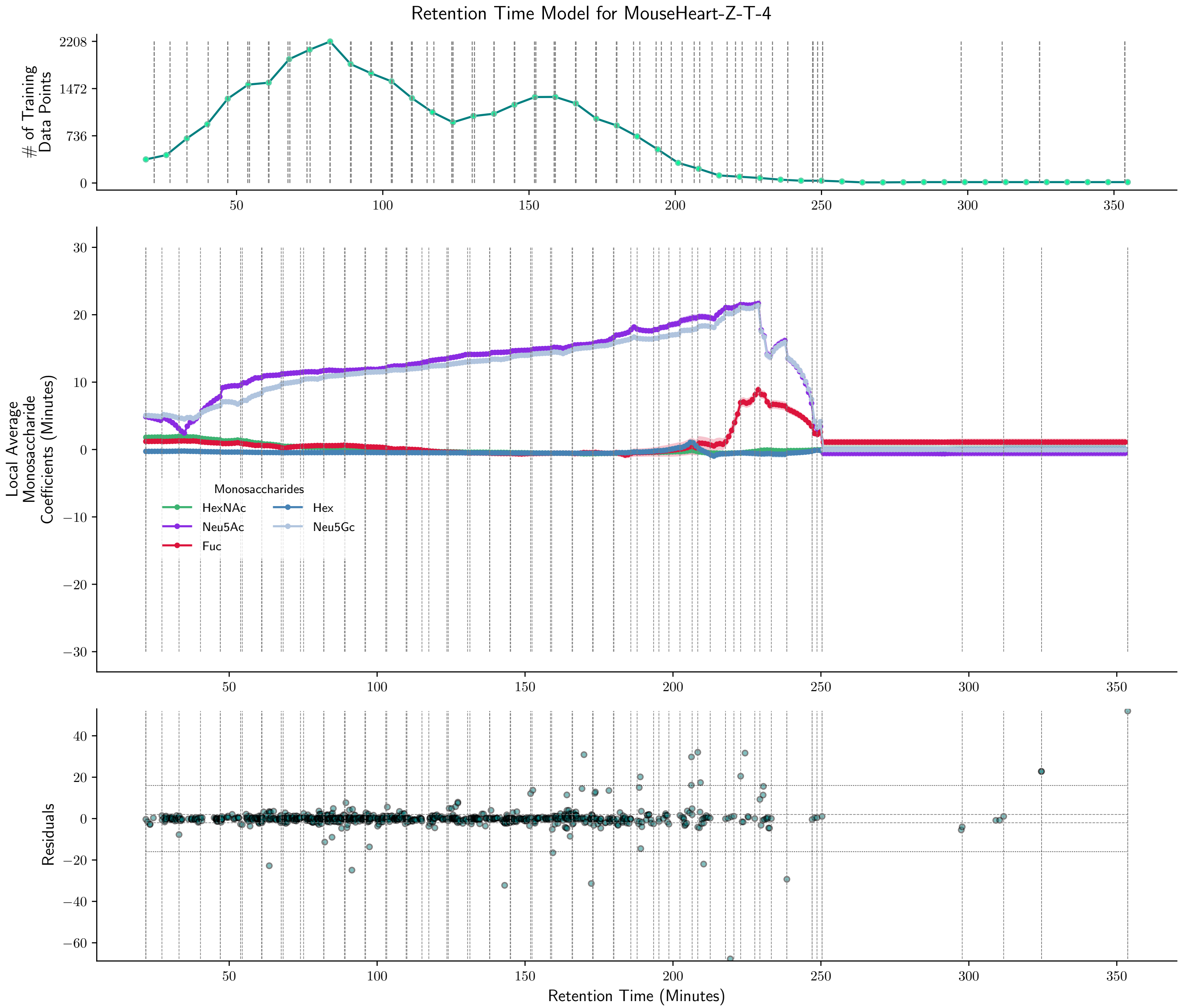

Figure 25: Extended retention time figure for Mouse Heart4. Extended retention time figure for Mouse Heart4

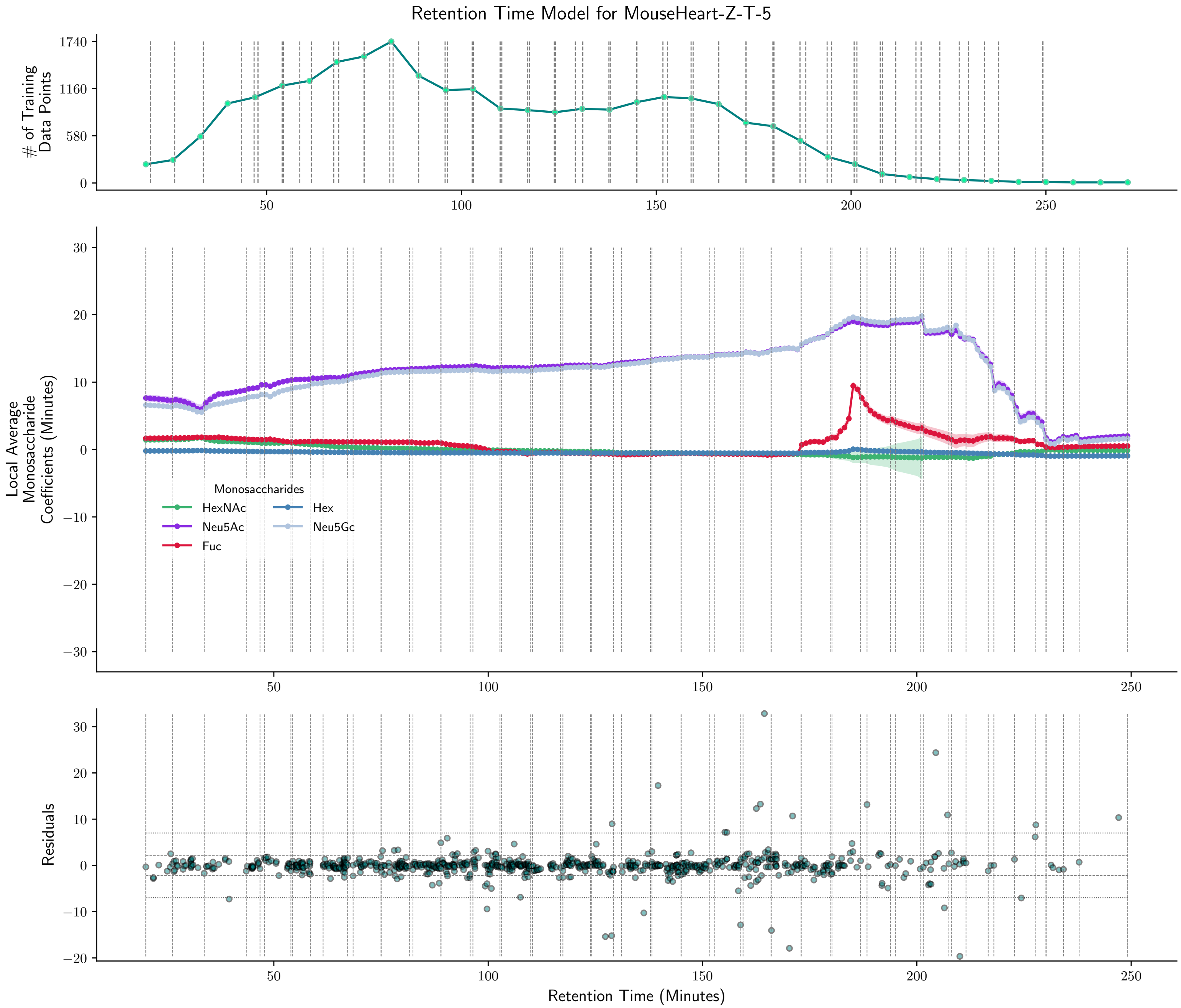

Figure 26: Extended retention time figure for Mouse Heart5. Extended retention time figure for Mouse Heart5

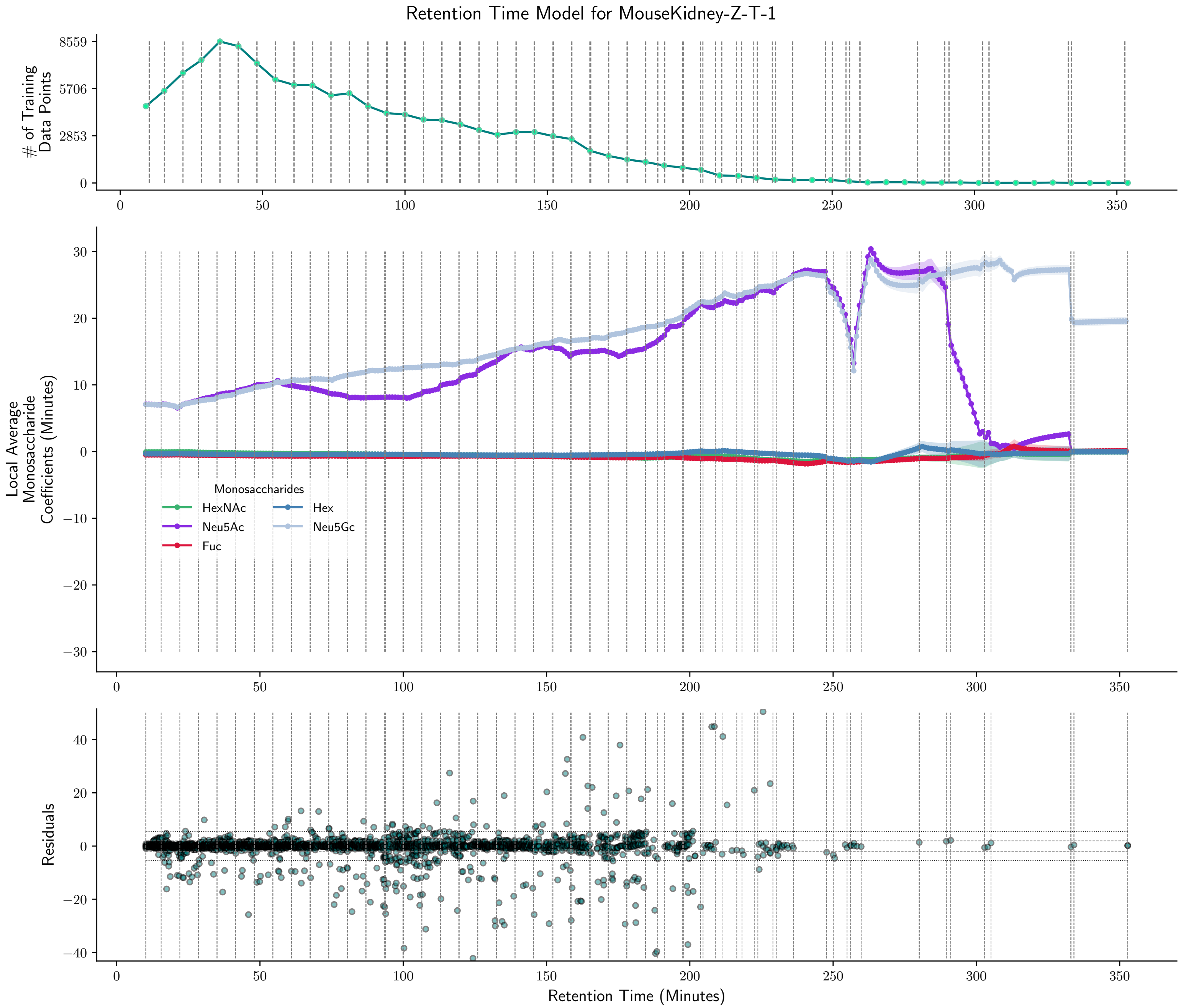

Figure 27: Extended retention time figure for Mouse Kidney1. Extended retention time figure for Mouse Kidney1

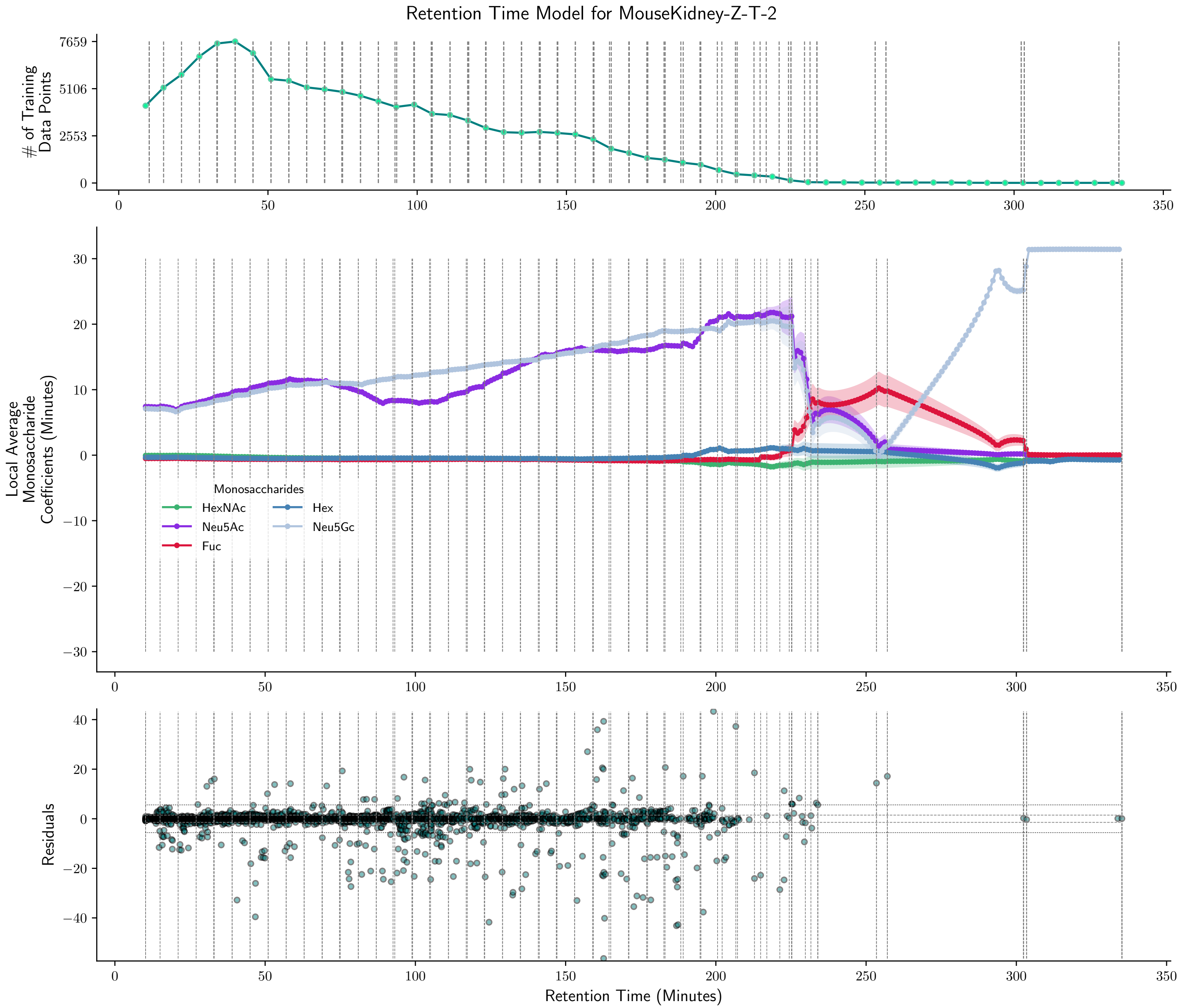

Figure 28: Extended retention time figure for Mouse Kidney2. Extended retention time figure for Mouse Kidney2

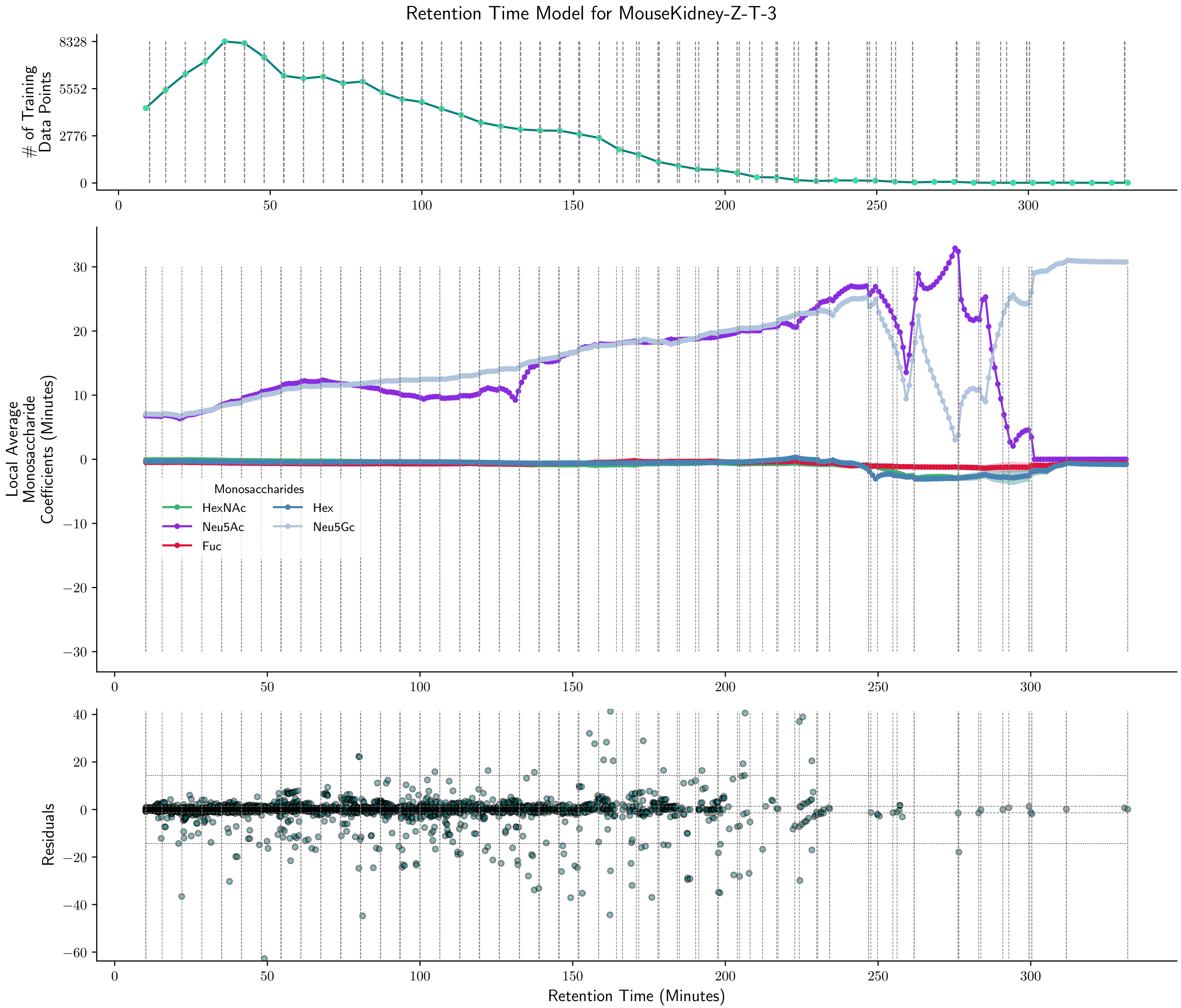

Figure 29: Extended retention time figure for Mouse Kidney3. Extended retention time figure for Mouse Kidney3

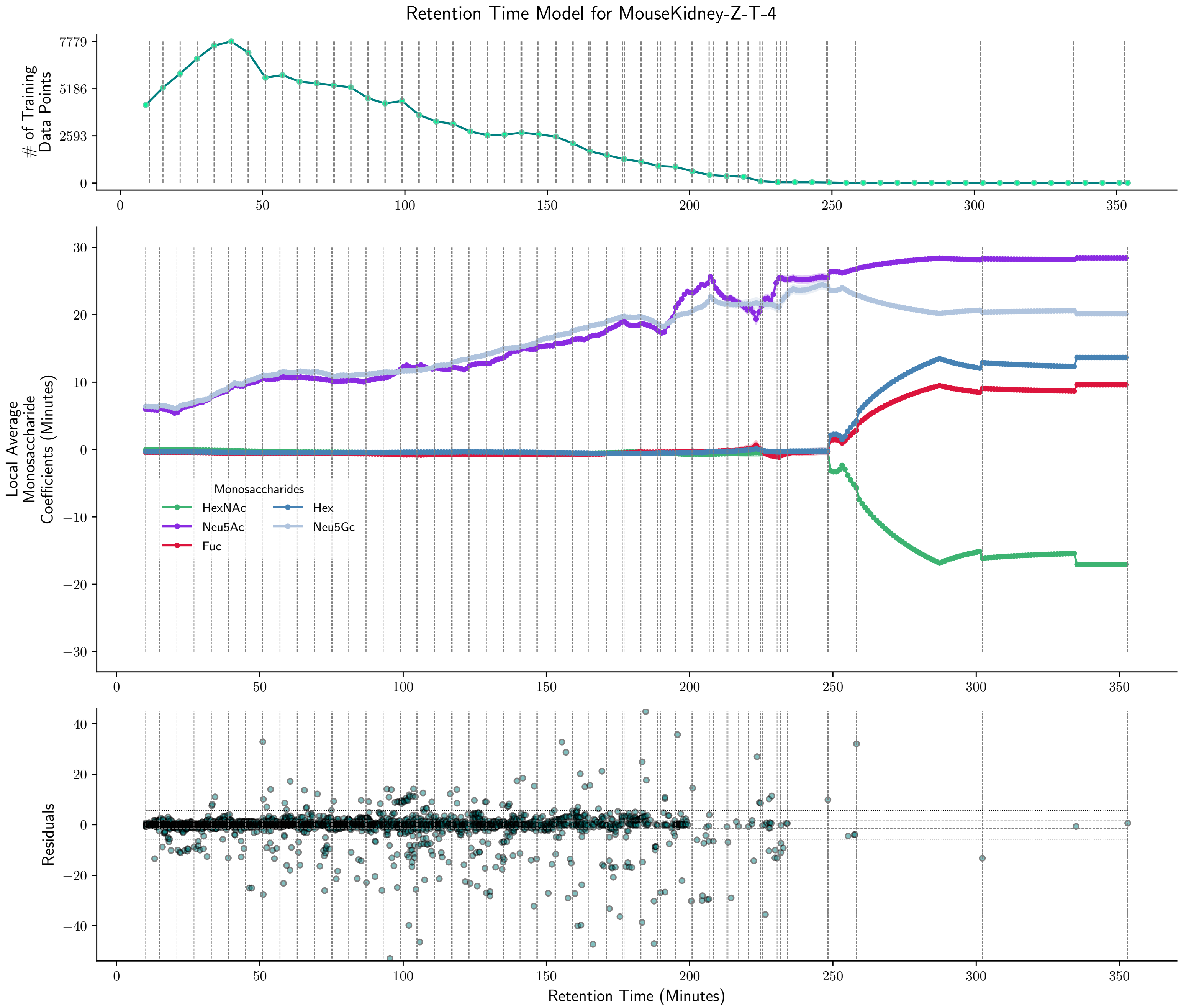

Figure 30: Extended retention time figure for Mouse Kidney4. Extended retention time figure for Mouse Kidney4

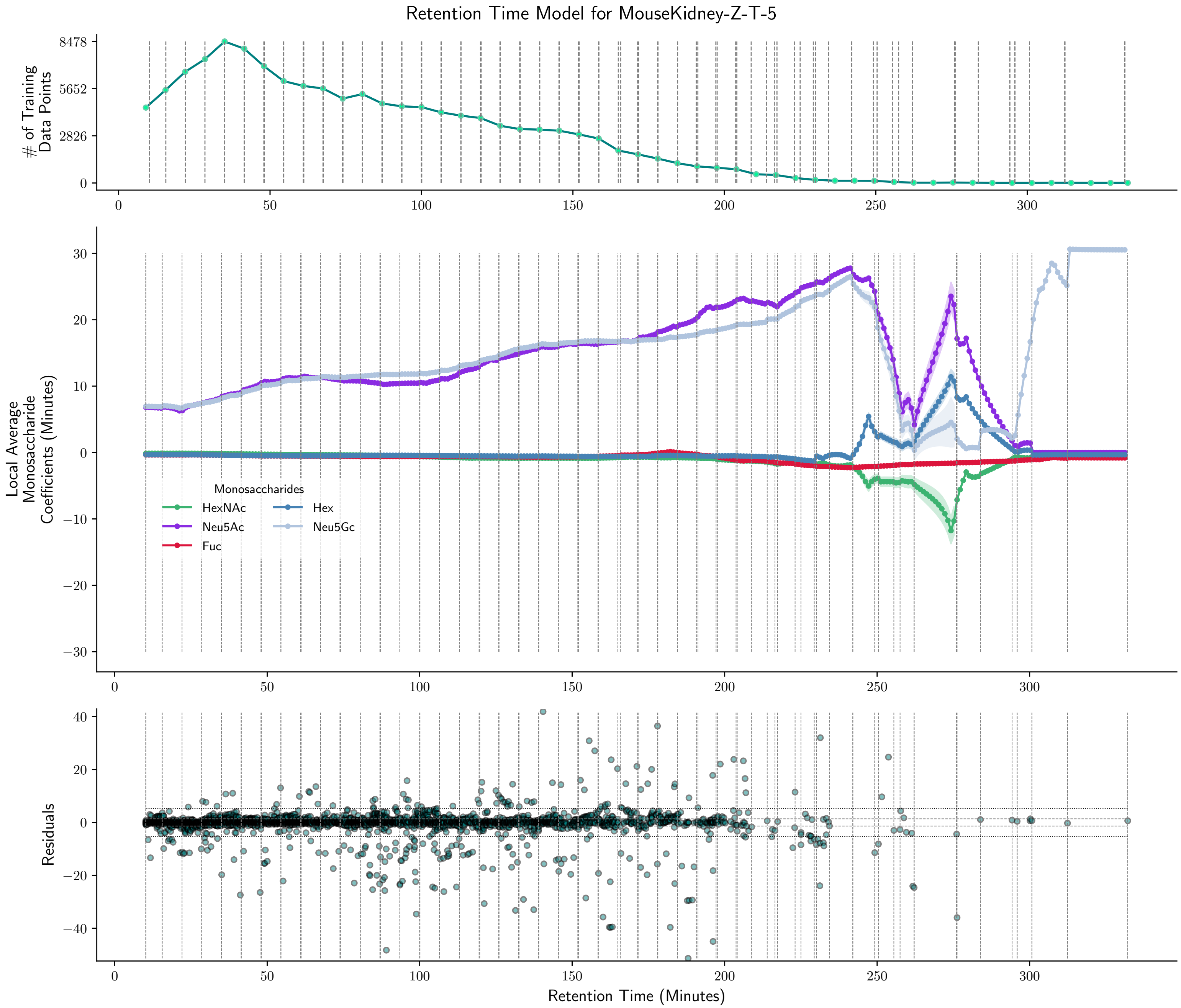

Figure 31: Extended retention time figure for Mouse Kidney5. Extended retention time figure for Mouse Kidney5

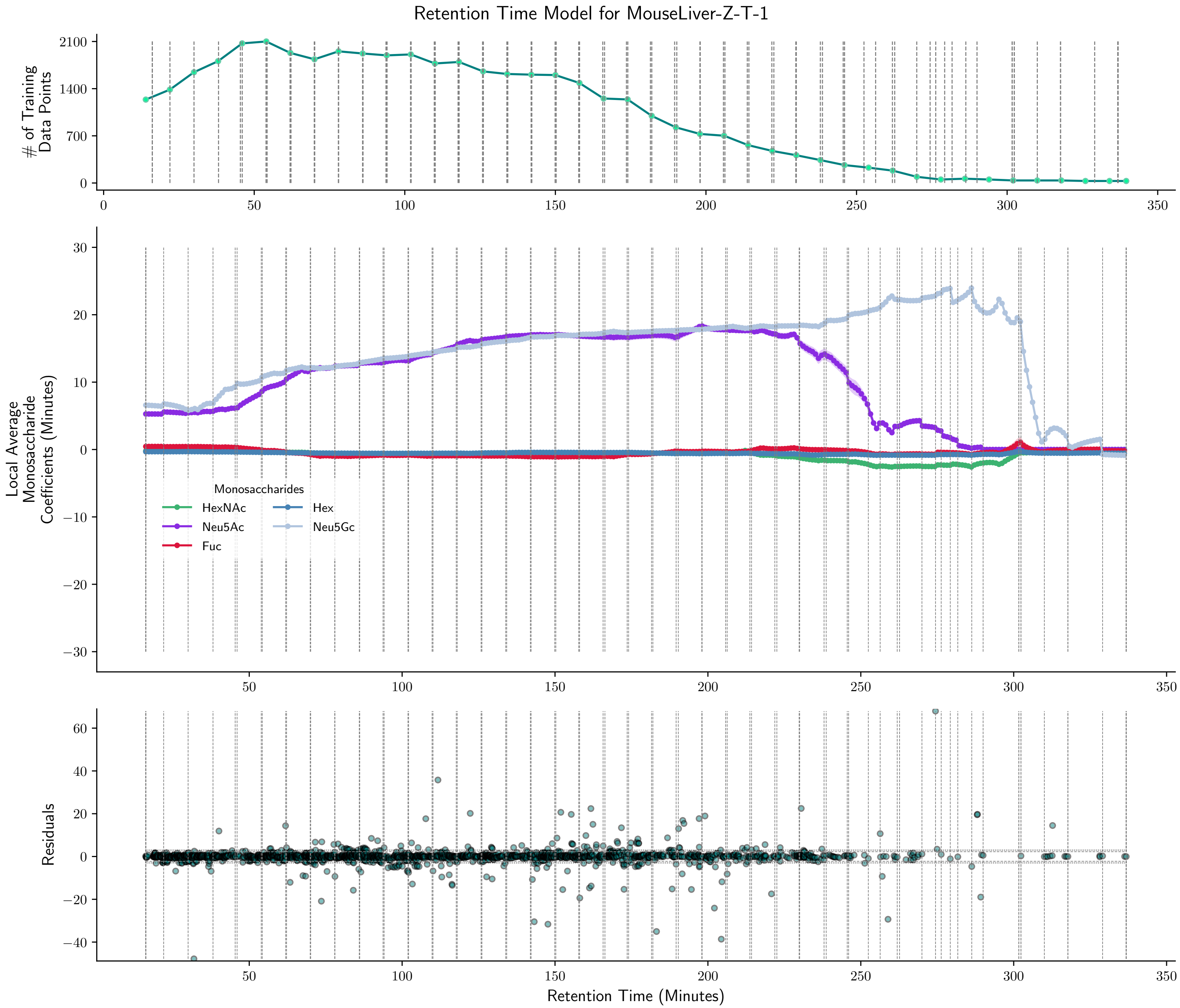

Figure 32: Extended retention time figure for Mouse Liver1. Extended retention time figure for Mouse Liver1

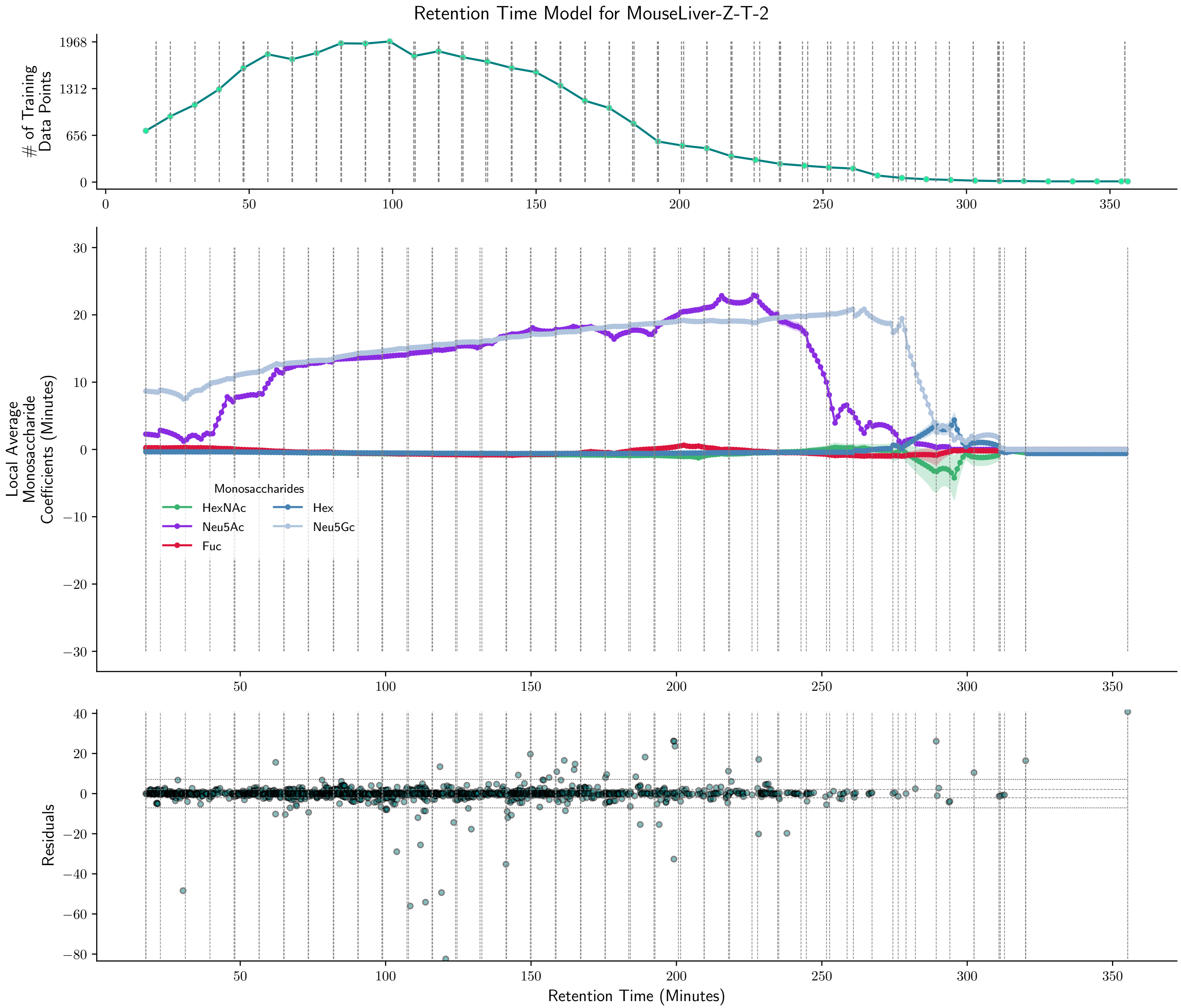

Figure 33: Extended retention time figure for Mouse Liver2. Extended retention time figure for Mouse Liver2

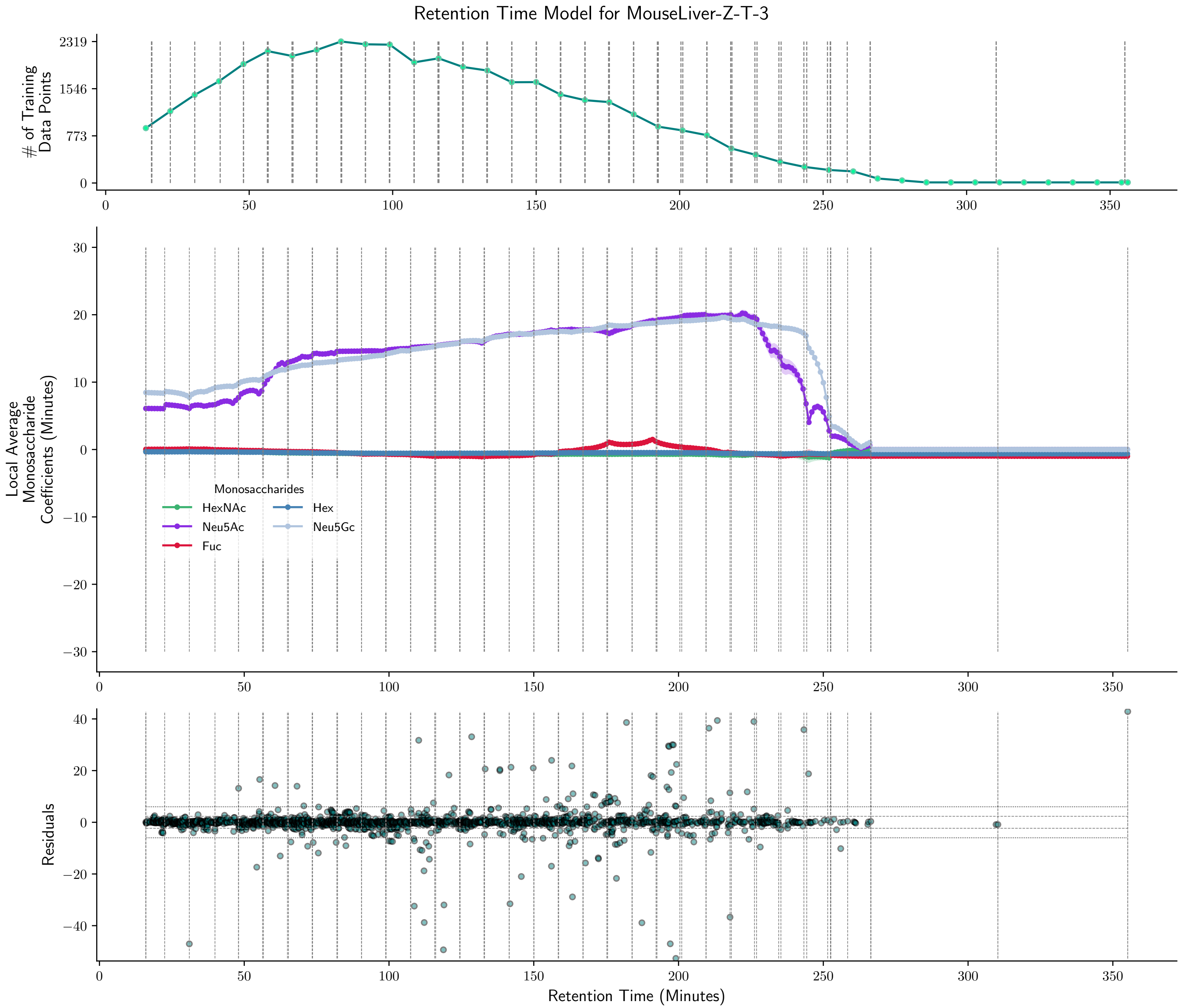

Figure 34: Extended retention time figure for Mouse Liver3. Extended retention time figure for Mouse Liver3

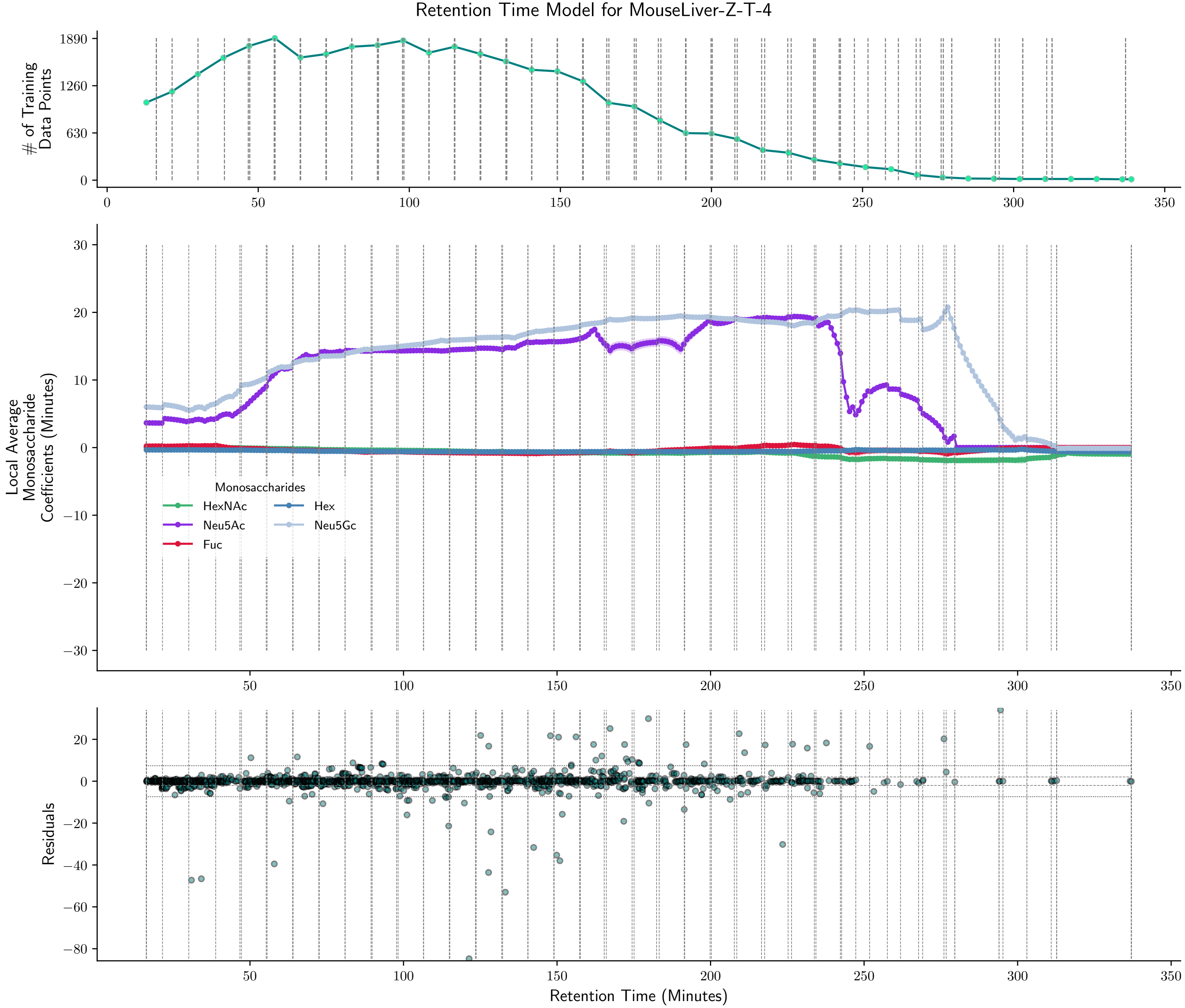

Figure 35: Extended retention time figure for Mouse Liver4. Extended retention time figure for Mouse Liver4

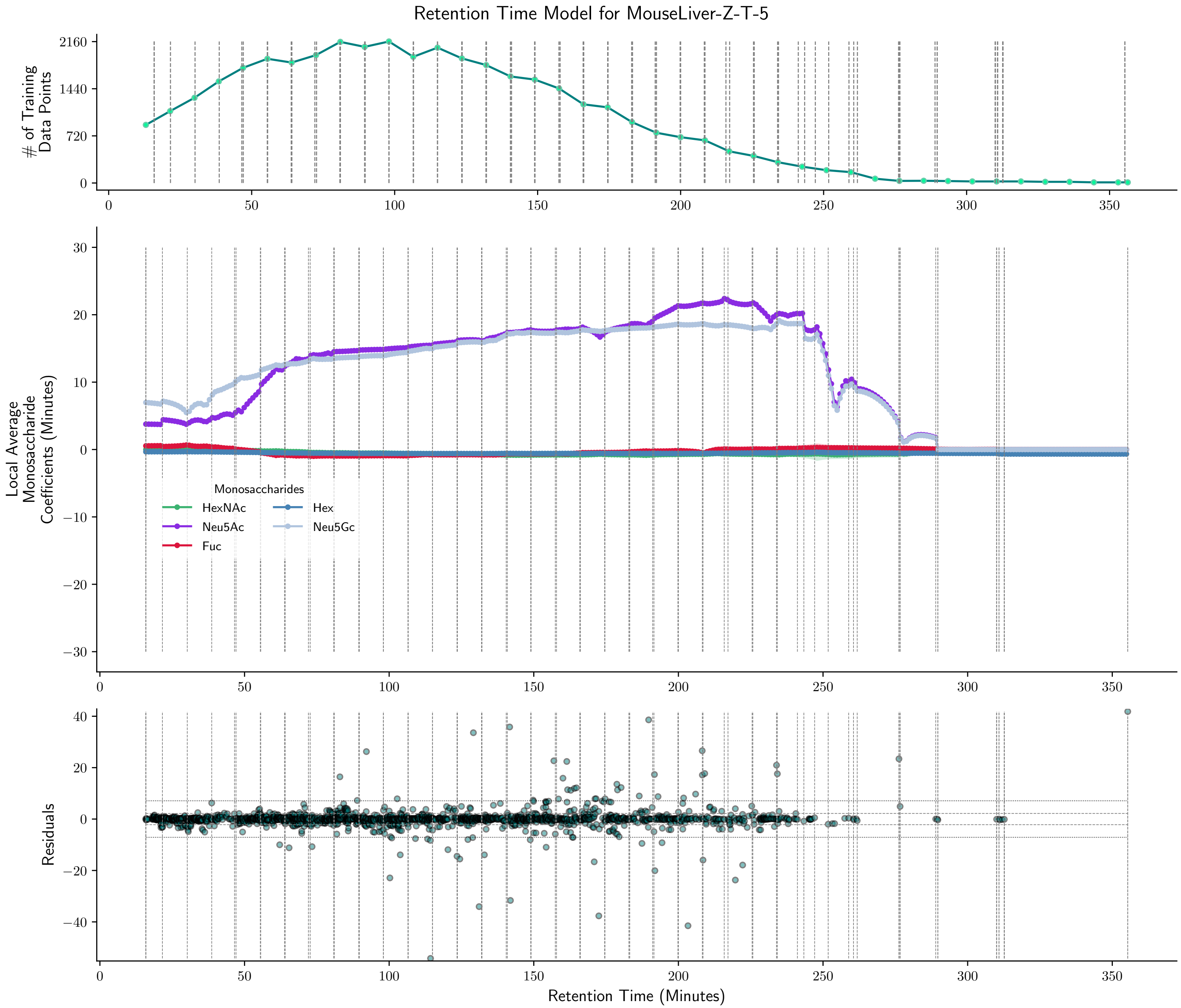

Figure 36: Extended retention time figure for Mouse Liver5. Extended retention time figure for Mouse Liver5

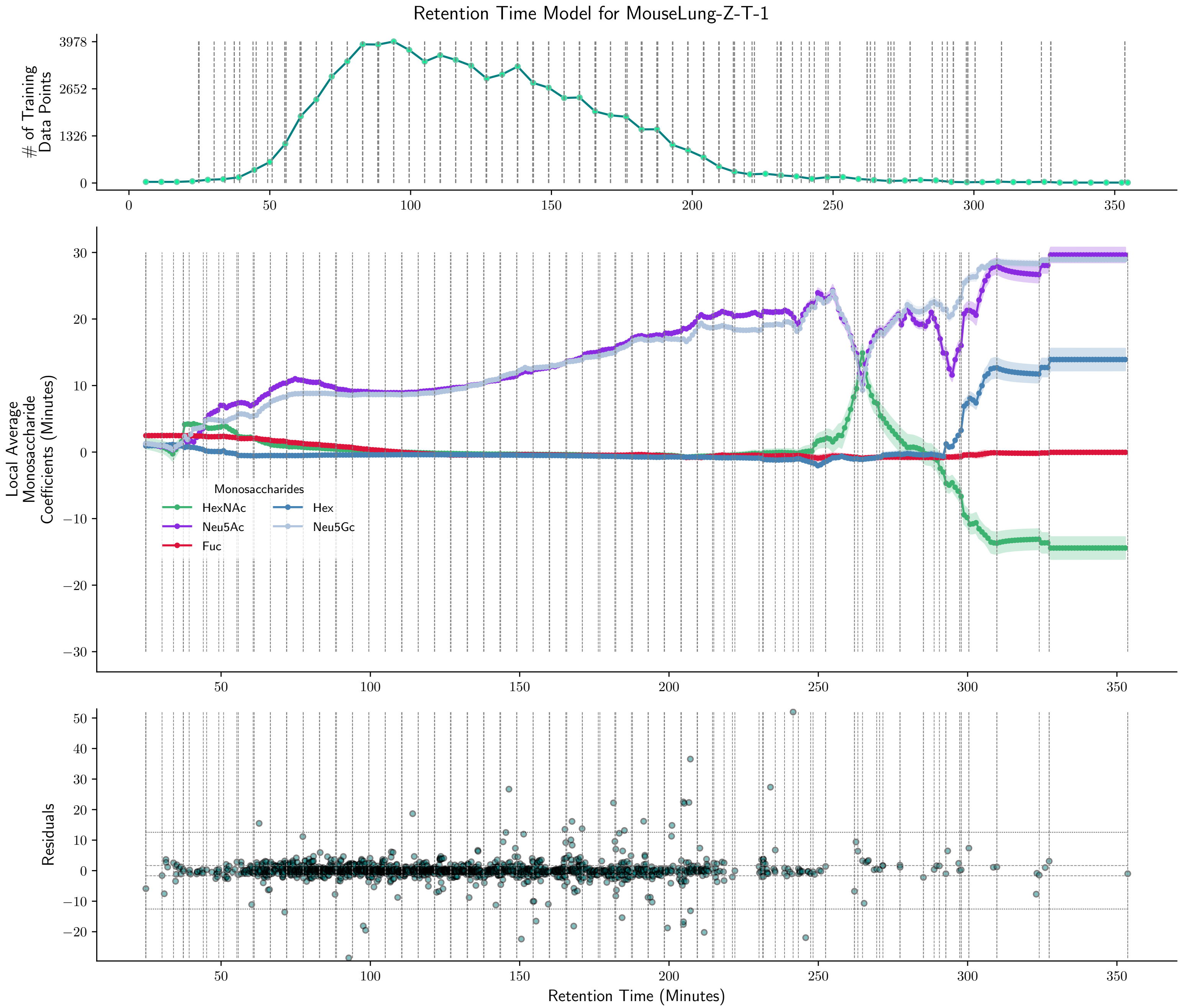

Figure 37: Extended retention time figure for Mouse Lung1. Extended retention time figure for Mouse Lung1

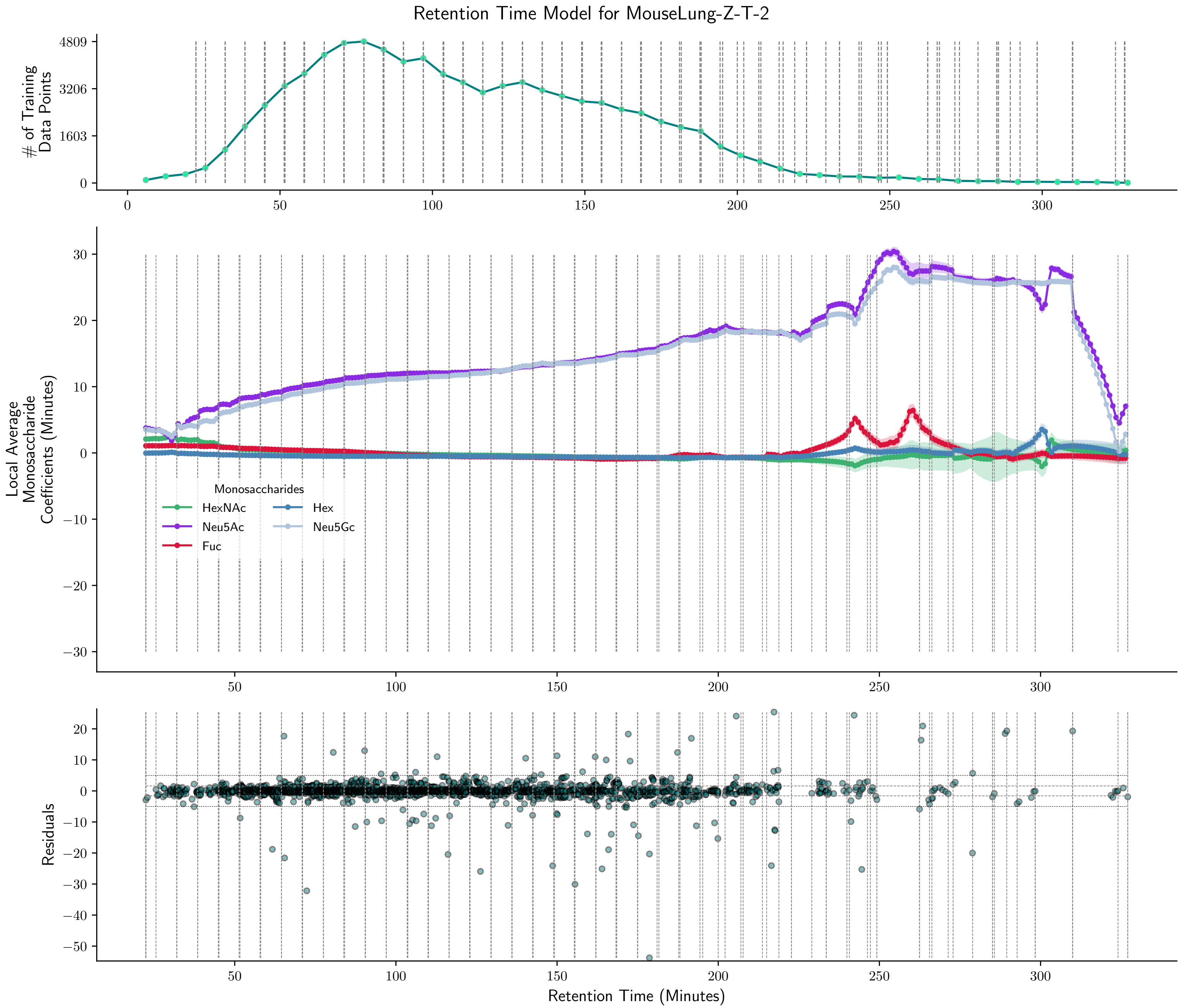

Figure 38: Extended retention time figure for Mouse Lung2. Extended retention time figure for Mouse Lung2

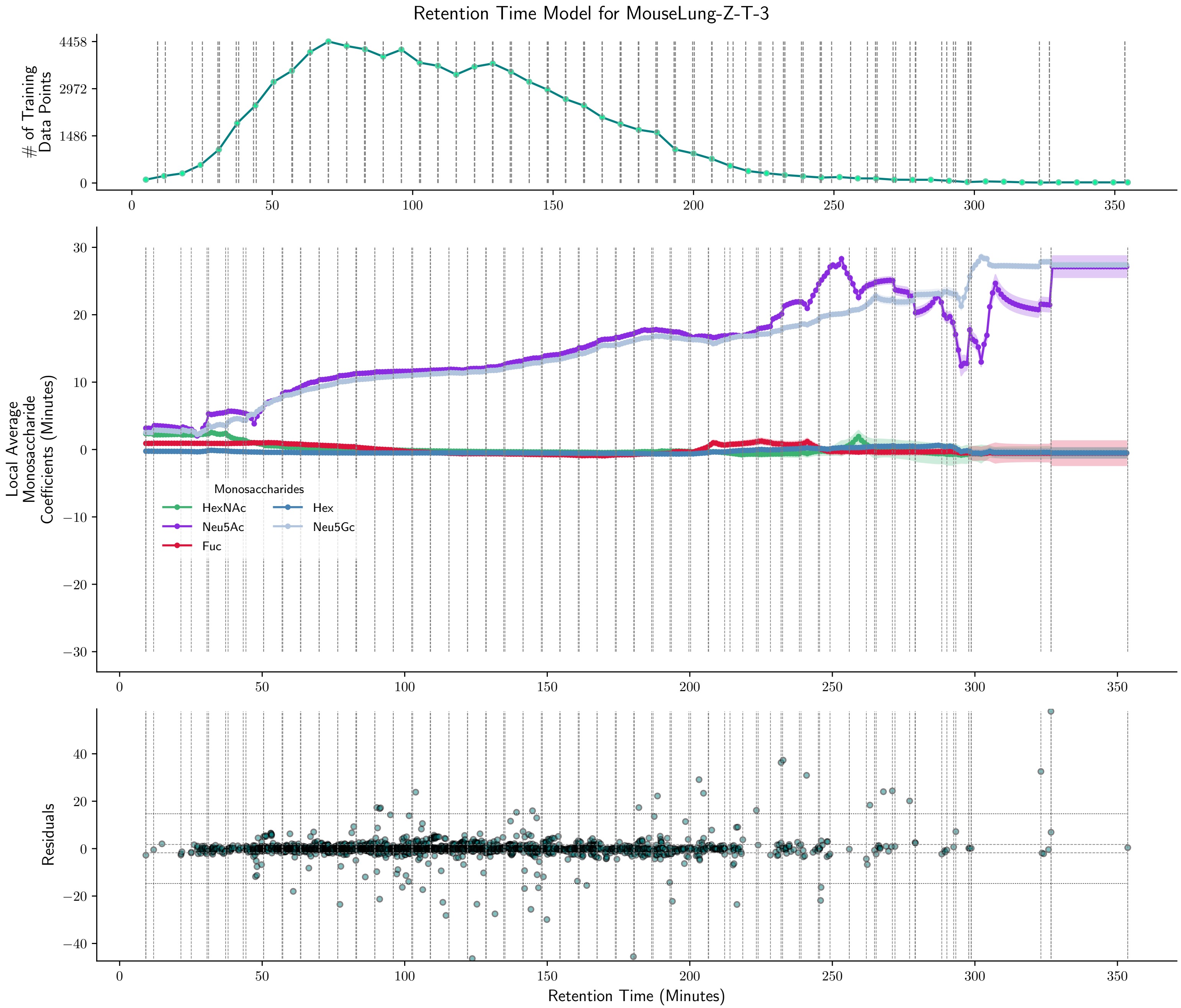

Figure 39: Extended retention time figure for Mouse Lung3. Extended retention time figure for Mouse Lung3

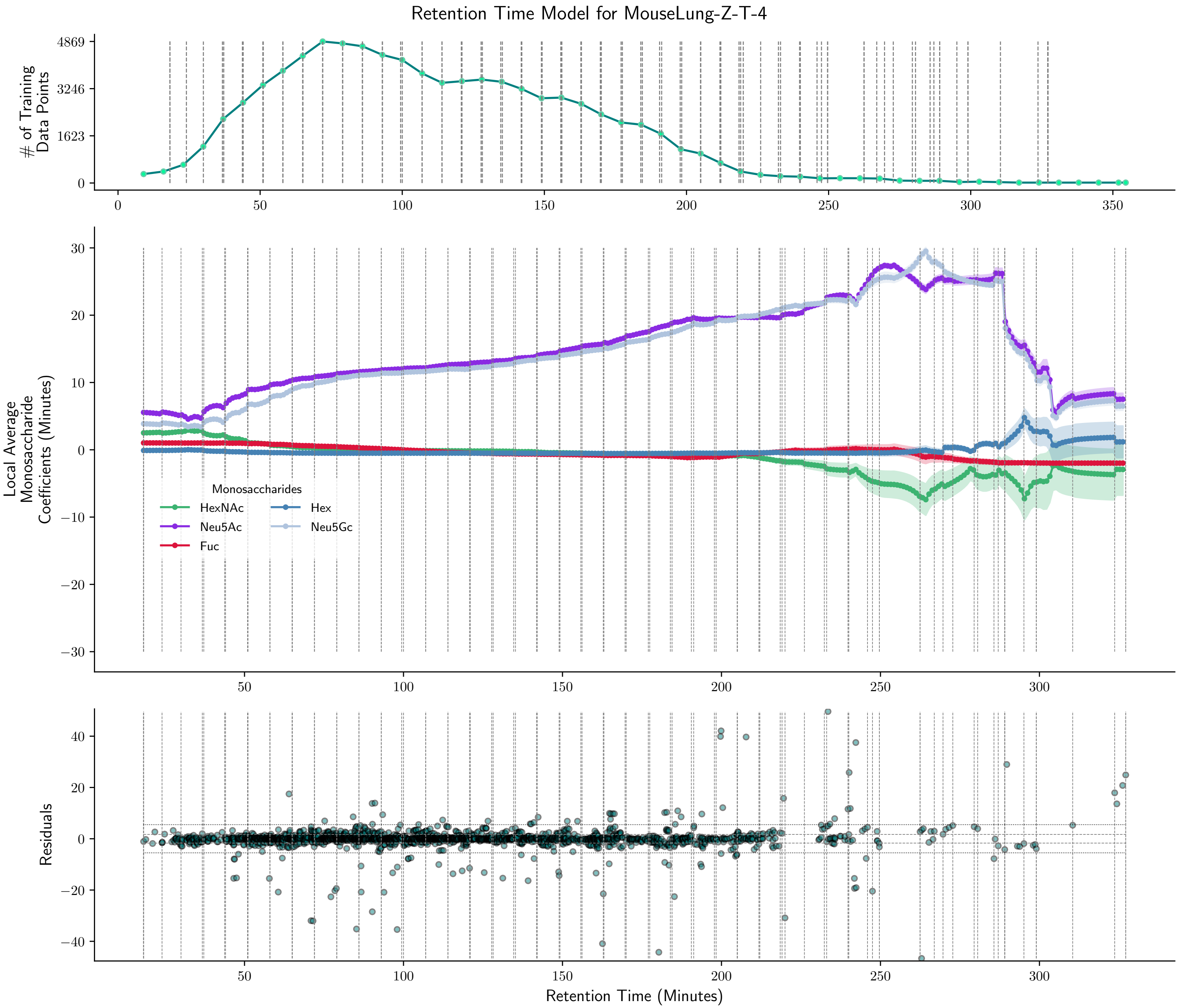

Figure 40: Extended retention time figure for Mouse Lung4. Extended retention time figure for Mouse Lung4

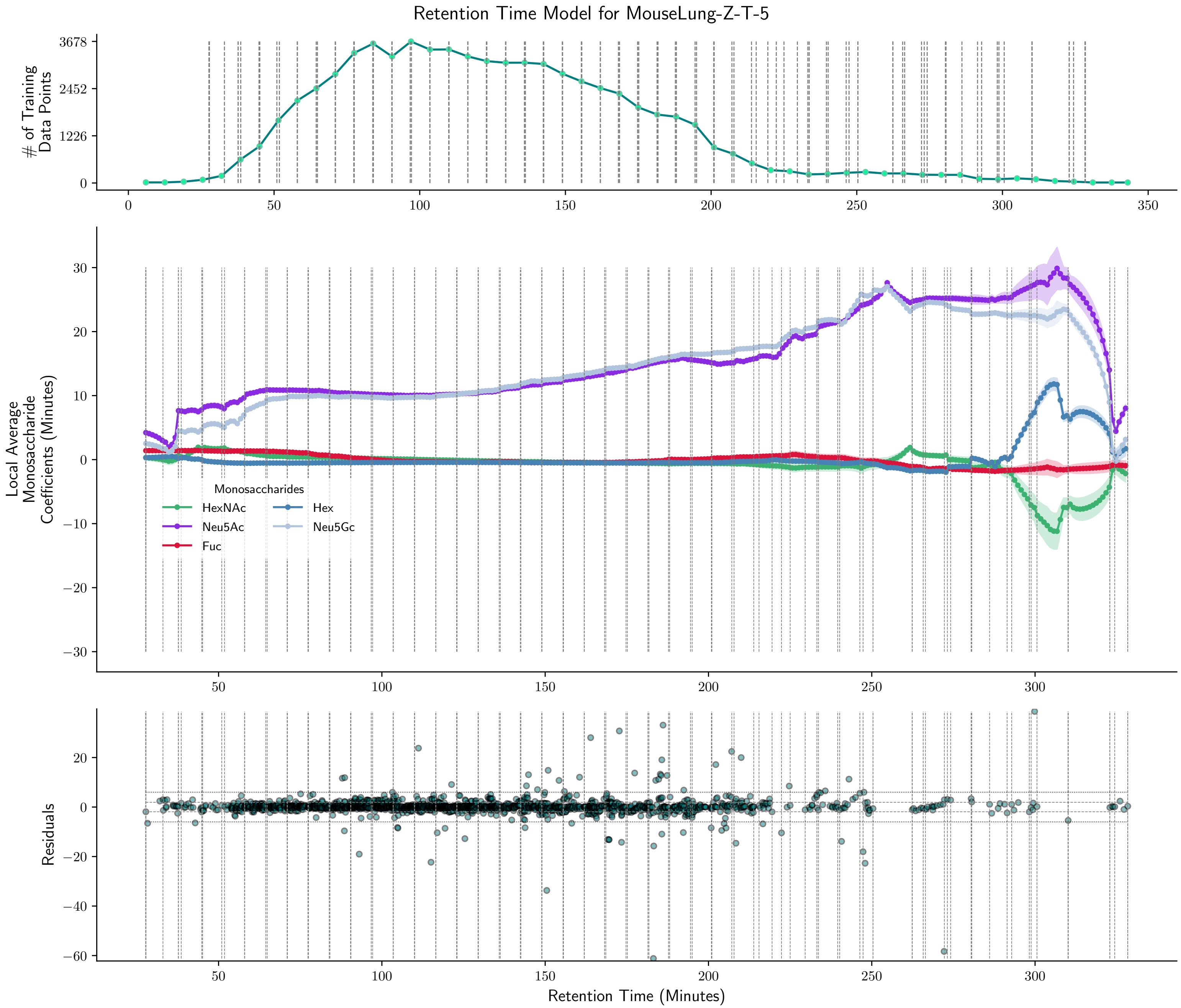

Figure 41: Extended retention time figure for Mouse Lung5. Extended retention time figure for Mouse Lung5

2 Supplementary Methods

2.1 Adduct Deconvolution and Retention Time Modeling

It is well understood that there are pervasive mass ambiguities in glycomics and glycoproteomics, some of which cannot reasonably be distinguished from a tandem mass spectrum alone Klein and Zaia (2020). To protect any models learned from the final output of the identification process from mis-assigned glycans, we need to correct these ambiguities.

2.1.1 Initial Adduct Deconvolution

We extended the method previously described in Klein and Zaia (2020) to model glycopeptide retention time integrated directly into the identification pipeline. After we assign chromatogram identifications, if a chromatogram was assigned an adducted glycopeptide g_a, if it does not overlap in time the un-adducted version g_u, the we re-assign the chromatogram to the next-best un-adducted glycopeptide g'_u barring the next-best un-adducted solution explaining less than 60% of the signal explained by the adducted solution on at least half of the supporting spectrum matches or failing to pass the required FDR threshold. After this initial deconvolution step, we merge all overlapping chromatograms for the same glycopeptide g regardless of their adduction state.

2.1.2 Retention Time Modeling

After initial adduct deconvolution and chromatographic peak shape scoring, we separate identified chromatograms into three bins, primary chromatograms $\mathbf{g}$ which are the most abundant chromatographic feature for each glycopeptide, secondary chromatograms $\mathbf{g}_{secondary}$ which are less abundant or which do not pass a chromatographic peak shape score of 6, and orphans which do not map to a chromatogram, $\mathbf{g}_{orphan}$. We will use g to denote both a glycopeptide identification and its representation as a feature vector containing an indicator variable for its peptide backbone sequence as well as ordinal values representing the count of each type of monosaccharide present in its glycan composition.

From $\mathbf{g}$, we select the subset which are both un-adducted and adducted in at least one form for the initial training set $\mathbf{g}_{\text{train},\mathbf{0}}$ to begin iteratively fitting a set of weighted linear models over overlapping retention time intervals. We include an initial weight vector $\mathbf{w} = \frac{1}{\text{abundance}(\mathbf{g})}$ which is updated iteratively.

Retention Time Bin Construction To determine retention time bin width for a given training set $\mathbf{g}$, we compute δ_{mono} Eq. S1, computing the absolute difference in apex retention time $RT(g)$ for each pair of glycopeptides which share the same peptide sequence and whose glycan compositions differ by only one mono monosaccharide, for each monosaccharide across all glycans in the training set, the overall median change in retention time caused by that monosaccharide. These are used to compute a retention time bin width Eq. S2. Next, we create a grid over retention time starting from the earliest time point $\min RT(\mathbf{g}) - 1$ to $\max RT(\mathbf{g}) + 1$, rounded down, spaced $\frac{\text{bin width}(\mathbf{g})}{2}$ minutes apart to be the centers of our retention time bins $\mathbf{b}$ with boundaries $b_i \pm \text{bin width}(\mathbf{g})$ from the centers. For each bin, we add members of $\mathbf{g}$ to $\mathbf{g}_{b_i}$ if their apex retention time $RT(g)$ falls within the bin's boundaries.

$$\delta_{\text{mono}}(\mathbf{g}) = \text{median}\left\{|RT(g_i) - RT(g_j)| \mid |\text{glycan diff}(g_i, g_j)| = 1_{\text{mono}};\ \text{peptide}(g_i) = \text{peptide}(g_j)\right\} \tag{S1}$$

$$\text{bin width}(\mathbf{g}) = \max_{\text{mono}\in\text{monosaccharides}(\mathbf{g})} \delta_{\text{mono}}(\mathbf{g}) \tag{S2}$$

Outlier Removal For each bin in $\mathbf{b}$, we pre-process the glycopeptides in each bin, for each peptide backbone, for each pairwise permutation of glycopeptides in $\mathbf{g}_{b_i}$ whose glycan compositions that differ by no more than 3 monosaccharide units, we create an observation $\hat{\mathbf{g}}_{b_i}$ where $RT(\hat{g}_k) = RT(g_i) - RT(g_j)$, whose peptide sequence is the shared peptide sequence and the glycan composition is $\text{glycan diff}(g_i, g_j)$ (the signed difference in count between g_i and g_j for each monosaccharide to be covered by the model), whose abundance weight is $\text{abundance}(\hat{g}_k) = \sqrt{\text{abundance}(g_i) \times \text{abundance}(g_j)}$ and whose weight $\hat{w}_k = \sum_{d \in \text{glycan diff}(g_i, g_j)} |d|^4 + (w_i + w_j)$. As each pair shares the same peptide backbone, we assume the peptide contribution is effectively removed from $RT(\hat{\mathbf{g}})$. For each bin's $\hat{\mathbf{g}}$ we group each synthetic feature by glycan composition and compute the mean and standard deviation of $\hat{\mathbf{g}}_{\text{glycan composition}}$ weighted by abundance, discarding any entry which is more than 2.5 standard deviations away from the group mean. This filtered set is what is referred by $\hat{\mathbf{g}}_{b_i}$ going forwards.

Fit First Linear Model For each bin in $\mathbf{b}$, we fit a weighted linear regression model using L_2 regularization and coefficients $\boldsymbol{\beta}_{b_i}$, variance-covariance matrix $\boldsymbol{\Sigma}_{b_i}$, degrees of freedom d_{b_i} and residual sum of squares RSS_{b_i} over all the peptide backbones and monosaccharides in $\hat{\mathbf{g}}_{b_i}$, which for the datasets considered here takes the form $RT_i \sim I(\text{Peptide}_i) + \text{Hex}_i + \text{HexNAc}_i + \text{dHex}_i + \text{NeuAc}_i + \text{NeuGc}_i$, denoted as β_{b_i}. The regularization weights are 0.01 for all monosaccharide coefficients and 0 for all other features. After fitting the model on the synthetic "relative" observations in $\hat{\mathbf{g}}_{b_i}$ we estimate a secondary peptide offsets for each distinct peptide backbone in $\hat{\mathbf{g}}_{b_i}$ (Eq. S3). The final predicted retention time for bin b_i is given in Eq. S4.

$$\text{offset}_{\text{peptide}_i, b_i} = \frac{1}{\sum_{\substack{\text{peptide}(g_i)=\text{peptide}_i,\\ g_i \in \hat{\mathbf{g}}_{b_i}}} w_i} \sum_{\substack{\text{peptide}(g_i)=\text{peptide}_i,\\ g_i \in \hat{\mathbf{g}}_{b_i}}} \boldsymbol{\beta}_{b_i} g_i \times w_i \tag{S3}$$

$$\widehat{RT}_{b_i}(g_i) = \boldsymbol{\beta}_{b_i} g_i + \text{offset}_{\text{peptide}(g_i), b_i} \tag{S4}$$

Iterative Model Updates After the initial models on $\mathbf{b}$ are fit on $\mathbf{g}_{\text{train},\mathbf{0}}$, we begin iteratively expanding the training dataset and updating the models. With the initial fitted model, we compute weighted coverage for each glycopeptide in $\mathbf{g}$ using Eq. S7. We define a coverage threshold k_j starting from 1 descending to 0.55 by steps of 0.05 over 10 additional iterations, denoting the set of training instances passing the coverage threshold k_j as $\mathbf{g}_{train,j}$.

$$\text{centroid}(\mathrm{b_i}) = \sum_{g \in \mathbf{g}_{b_i}} \frac{1}{\text{abundance}(g)} \sum_{g \in \mathbf{g}_{b_i}} RT(g) \times \text{abundance}(g) \tag{S5}$$

$$\text{weight}(g, \mathbf{b}) = \left\{ |(\text{centroid}(b_i) - RT(g)| + 1)^{-1} \mid b_i \in \mathbf{b} \right\} \tag{S6}$$

$$\text{coverage}(g) = \sum_{v \in \text{weight}(g, \mathbf{b})} \frac{1}{v} \sum_{\substack{b \in \mathbf{b}\\ v \in \text{weight}(g, \mathbf{b})}} v \times \widehat{RT}_{b_i}(g) \tag{S7}$$

During each iteration j, after the set of glycopeptides in $\mathbf{g}_{train,j}$, instances are re-weighted based upon how well they fit the ensemble model. We denote compute a weighted average of prediction intervals at $\alpha = 0.01$ over each sub-model in the ensemble as in Eq. S10 and compute the 25th and 75th percentiles of the width of the interval to estimate a minimum and maximum bound in Eq. S11. We denote the interval score (Eq. S12) as a value in $[0, 1]$ where 1 reflects a perfect match to the model expectations, and we update the weight of each glycopeptide g_i to the sum of its total abundance plus $\max(\text{interval score}(g_i), 0.01)$.

$$\widehat{RT}(g) = \sum_{v \in \text{weight}(g,\mathbf{b})} \frac{1}{v} \sum_{\substack{b \in \mathbf{b} \\ v \in \text{weight}(g,\mathbf{b})}} \widehat{RT}_{b_i}(g) \times v \tag{S8}$$

$$PI_{b_i}(g, \alpha) = \widehat{RT}_{b_i} \pm \bar{T}(\alpha/2, d_{b_i}) \times \sqrt{\frac{RSS_{b_i}}{d_{b_i}} \times (1 + g\mathbf{\Sigma}_{b_i} g^t)} \tag{S9}$$

$$PI(g, \alpha) = \sum_{v \in \text{weight}(g,\mathbf{b})} \frac{1}{v} \sum_{\substack{b \in \mathbf{b} \\ v \in \text{weight}(g,\mathbf{b})}} PI_{b_i}(g, \alpha) \times v \tag{S10}$$

$$\text{prediction width}(\alpha) = \text{percentile}\left(\left\{PI(g, \alpha)_{\text{upper}} - \widehat{RT}(g) \mid g \in \mathbf{g}\right\}, [25, 75]\right) \tag{S11}$$

$$\text{interval score}(g, \alpha) = \max\left(1 - \frac{|RT(g) - \widehat{RT}(g)|}{h(g, \alpha)}, 0.0\right) \tag{S12}$$

$$h(g, \alpha) = \text{clamp}\left(PI(g, \alpha)_{\text{upper}}, \text{prediction width}(\alpha)\right) + \text{interval padding} \tag{S13}$$

where the "interval padding" term in Eq. S13 is 0 initially, re-estimated later from the mean squared error of the final model and the residual false discovery rate. After each iteration, all observations in $\mathbf{g}_{train,j}$ are tested for revision, as described in section 2.1.2, potentially updating their glycan component.

After 10 iterations, there is an "open update" round where the coverage threshold is removed, and glycopeptides whose peptide backbone is present more than twice are also considered during model fitting to allow uncovered peptide backbones to be incorporated into the model and may be revised. Then another 10 iterations of refitting and glycan revision follows once again starting $k = 1$, reducing by 0.05 per iteration with a final "open update" round after that. We will refer to $\mathbf{g}_{train,22}$ as $\mathbf{g}_{trained}$.

Glycan Composition Revision After each iteration of model fitting, the retention time model ensemble is used to evaluate each glycopeptide against a set of glycan composition substitution rules used, shown in Table 4, though additional rules are available for phosphorylated or sulfated glycans. The revision that maximizes the interval score for a glycopeptide is selected, provided that the revision's score is greater than the original score by 0.35 or more, is predicted to be $\text{prediction width}(0.01)_{\text{lower}}$ or more separated from the original, does not result in the loss of an abundant oxonium ion (signal utilization after revision $\geq 90\%$ before) or a large reduction in total peptide+Y ion abundance (signal utilization after revision $\geq 85\%$ before), and that the resulting glycan composition is found in the glycan search space being considered.

Final Revision and Retention Time Error Bounds Calibration Via FDR Estimation After the final model is fit and observations are revised, the model may be usable, but it does not well calibrated to the variability found in larger datasets from chromatographic drift, inconsistent peak shapes or peptide-specific effects. We update the interval padding term of Eq. S13 to Eq. S14 and perform a final round of glycan revision with a small increase in sensitivity.

Glycan Component	Alternate	Reverse-able
Hex 1, dHex 1	NeuAc 1, Ammonium	Yes
Hex 1, NeuAc 1	dHex 1, NeuGc 1	Yes
Hex 2	NeuGc 1, Ammonium	Yes
dHex 2	NeuAc 1	No
dHex 4	NeuAc 2	No
Hex 6	HexNAc 2, NeuAc 2, Ammonium	No
Hex 7	HexNAc 2, NeuAc 2, dHex 1, Ammonium	No

Table 4: The substitution rules used for the datasets described in this work.

$$\begin{aligned}\text{padding}_{mean}(\mathbf{g}) = |\mathbf{g}|^{-1} \times \\ \sum_{g \in \mathbf{g}} \min |RT(g) - \text{PI}(g, \alpha)| - \\ \left(\text{clamp}\left(PI(g,\alpha)_{\text{upper}},\ \text{prediction width}(0.01)\right) - \widehat{RT}(g)\right)\end{aligned} \tag{S14}$$

$$\text{resid}(\mathbf{g}) = RT(\mathbf{g}) - \widehat{RT}(\mathbf{g}) \tag{S15}$$

After the final revision, each glycopeptide g in $\mathbf{g}_{trained}$ that was revised from the original g^* in $\mathbf{g}_{train,0}$ value is re-tested to see if its original glycan composition would be a better fit using the final model compared to the intermediate model along the way. A revision is rejected if $|\text{interval score}(g, 0.01) - \text{interval score}(g^*, 0.01)| < 0.35$. If neither version of the glycopeptide is supported by the current model, the revision is rejected if $\frac{|RT(g) - \widehat{RT}(g)|}{|RT(g) - \widehat{RT}(g^*)|} \geq 0.5$.

To boost the sensitivity of the model without unduly reducing its specificity further and allow it to adapt to out-of-distribution observations, we compute the a retention time error model using a mixture model approach like the one used for false discovery rate estimation. For each revision rule used from Table 4 for each glycopeptide g in $\mathbf{g}_{trained}$, if the substituted glycan composition is found in the glycan search space, the substituted glycopeptide $g_{substituted}$ with the same retention time as g is added to $\mathbf{g}_{decoy\ substituted}$. We also generate a random permutation of $\mathbf{g}_{trained}$ shuffling the relationship between g and $RT(g)$ as $\mathbf{g}_{decoy\ permuted}$. We compute the residuals as in Eq. S15 for each of $\mathbf{g}_{trained}$, $\mathbf{g}_{decoy\ substituted}$, and $\mathbf{g}_{decoy\ permuted}$. We fit two mixtures of gaussian distributions to the combinations of $\text{resid}(\mathbf{g}_{trained})$ and each decoy group's residuals, permitting up to three distributions for the target component, denoting them $fmm_{substituted}$ and $fmm_{permuted}$. Each mixture maps retention time error to the posterior error probability of occurrence. This function is not monotonic, so we create a monotonic variant of it , $\overline{fmm}$, over a grid from 0 to the greatest magnitude retention time error and normalize it to be between 0 and 1, subtracted from 1 to make it always decreasing instead of always increasing. We pick between $\overline{fmm}_{substituted}$ and $\overline{fmm}_{permuted}$ which reaches 0.5 at the smaller retention time error. On average, $fmm_{permuted}$ overestimates uncertainty on large datasets but $fmm_{substituted}$ overestimates even more poorly on small or sparse datasets. The quantity $\text{padding}_{residual} = \min(x - \text{prediction width}(0.01)_{upper},$ bin width/2) where $\overline{fmm}(x) = 0.95$.

Secondary Revisions After calibrating the model, we return to $\mathbf{g}_{secondary}$ and $\mathbf{g}_{orphan}$, performing a single round of glycan revision on each collection of glycopeptide identifications. As orphans have no chromatogram mappings, we treat them as a single point chromatogram whose apex retention time is their scan time.

2.2 Glycan Composition Fragments and Indexing

GlycReSoft works with glycan compositions, not topologies or structures. This is because collisional dissociation does not provide the necessary cross-ring fragmentation product ions to reliably localize a monosaccharide to a specific branch of a glycan structure, and gas-phase rearrangement can shift the position of fucosylation. Generating fragments from a composition instead of a topology is more difficult without making some simplifying assumptions. GlycReSoft generates peptide+Y fragments for glycan compositions based upon the monosaccharide counts and the glycan class, e.g. *N*-glycosylation or *O*-glycosylation. The procedure used to generate fragments from a theoretical *N*-glycan composition is shown in Listing 1. It assumes that the chitobiose core is not labeled chemically and that no other monosaccharides appear in the expanded structure. For the majority of naturally occurring cases in mammalian samples, this is sufficient as sialic acids tend to be too labile to be included in peptide+Y ions. This procedure is not optimal because it generates some combinations of monosaccharides which do not make sense given canonical *N*-glycan biosynthesis. The version used in practice also handles xylosylation of the chitobiose core as found in plants, and a similar algorithm is used for mucin-type *O*-glycans, using the type 1 core as its foundation instead of the chitobiose core.

After enumerating fragments from each glycan composition, a complement index is constructable following the same procedure described in Zeng *et al.* (2021). Scoring matches against the index also requires using the glycan coverage approximation described in Section 4.2, but otherwise functions as normal.

```
Data: Glycan Composition Mapping Monosaccharide To Count G
Result: List of Glycan Fragments F Possible From G
F ← ∅;
/* The total amount of each monosaccharide in the aggregate glycan
   composition attached to this peptide                              */
n_HexNAc ← G[HexNAc];
n_Hex ← G[Hex];
n_dHex ← G[dHex];
c_HexNAc ← min(n_HexNAc + 1, 3) ;                 // Detect truncated cores
c_Hex ← min(n_Hex + 1, 4);
for i_HexNAc ∈ [0, c_HexNAc) do             // The HexNAc of the chitobiose core
    if i_HexNAc = 0 then
        Append(F, {}) ;                                // The empty fragment
    else if i_HexNAc = 1 then
        f ← {HexNAc : i_HexNAc, is_core : true};
        Append(F, f);
        /* If there are any dHex in the composition, copy the current fragment
           and add a dHex to it and add that to the set of fragments,
           incrementally until all dHexs are used                         */
        if n_dHex > 0 then Append(F, dHexShift(f, n_dHex)) ;
    else              // Now Hexose can begin to appear from the chitobiose core
        f ← {HexNAc : i_HexNAc,is_core:true};
        Append(F, f);
        if n_dHex > 0 then
            Append(F, dHexShift(f, n_dHex));
        for i_Hex ∈ [1, c_Hex) do
            f ← {HexNAc : i_HexNAc, Hex : i_Hex, is_core : true};
            Append(F, f);
            if n_dHex > 0 then Append(F, dHexShift(f, n_dHex)) ;
            if i_Hex = 3 &n_HexNAc > 2 then       // Extend beyond the conserved core
                for j_HexNAc ∈ [0, n_HexNAc − i_HexNAc] do
                    f ← {HexNAc : i_HexNAc + j_HexNAc, Hex : i_Hex, is_core : false};
                    Append(F, f);
                    if n_dHex > 0 then Append(F, dHexShift(f, n_dHex)) ;
                    if n_Hex > 3 then
                        for j_Hex ∈ [1, n_Hex − i_Hex] do
                            f ← {HexNAc : i_HexNAc + j_HexNAc, Hex : i_Hex + j_Hex, is_core : false};
                            Append(F, f);
                            if n_dHex > 0 then Append(F, dHexShift(f, n_dHex));
                        end
                end
        end
end
return F
```

Algorithm 1: *N*-Glycan Composition Fragment Generation

2.3 Observation Partitioning For Fragmentation Modeling

Peptide Length	
Min	Max
0	4
5	9
10	14
15	19
20	24
25	29
30	34
35	39
40	44
45	49

(a)

Proton Mobility	
$\lvert H, K, R \rvert$ - Precursor z	Label
> 0	Mobile
$= 0$	Partial
< 0	Immobile

(b)

Table 5: Glycopeptide Model Partitions. We partitioned glycopeptide spectrum matches from the training data into groups according to each of the ranges specified in Tables 5a, 5b, their precursor charge z and the type and number of occupied glycosylation sites, producing up to 150 theoretical partitions per glycosylation type.

2.4 Fragmentation Modeling Features

The set of features in Table 6 enumerate all the intensity-predicting features for our multinomial logistic regression model. The peptide b and y ion features are derived from earlier works by Frank (2009) and Zubarev *et al.* (2008), while the peptide+Y ion features are based upon the relationship between peptide backbone composition observed in Kolli *et al.* (2015) and Aboufazeli and Dodds (2018). While our model does not have the degrees of freedom to fit higher order features and distant relationships, it can capture main trends.

n-term pro	stub glycopeptide:is_glycosylated 2	stub glycopeptide:charge 1:glycan loss 4	stub glycopeptide:charge 3:glycan loss 17
n-term gly	stub glycopeptide:is_glycosylated 3	stub glycopeptide:charge 1:glycan loss 5	stub glycopeptide:charge 3:glycan loss 18
n-term ser_thr	stub glycopeptide:is_glycosylated 4	stub glycopeptide:charge 1:glycan loss 6	stub glycopeptide:charge 3:glycan loss 19
n-term leu_iso_val_ala	stub glycopeptide:is_glycosylated 5	stub glycopeptide:charge 1:glycan loss 7	stub glycopeptide:charge 3:glycan loss 20
n-term asn	stub glycopeptide:is_glycosylated 6	stub glycopeptide:charge 1:glycan loss 8	stub glycopeptide:charge 4:glycan loss 0
n-term his	stub glycopeptide:is_glycosylated 7	stub glycopeptide:charge 1:glycan loss 9	stub glycopeptide:charge 4:glycan loss 1
n-term arg_lys	stub glycopeptide:is_glycosylated 8	stub glycopeptide:charge 1:glycan loss 10	stub glycopeptide:charge 4:glycan loss 2
n-term x	stub glycopeptide:is_glycosylated 9	stub glycopeptide:charge 1:glycan loss 11	stub glycopeptide:charge 4:glycan loss 3
c-term pro	stub glycopeptide:is_glycosylated 10	stub glycopeptide:charge 1:glycan loss 12	stub glycopeptide:charge 4:glycan loss 4
c-term gly	stub glycopeptide:composition pro	stub glycopeptide:charge 1:glycan loss 13	stub glycopeptide:charge 4:glycan loss 5
c-term ser_thr	stub glycopeptide:composition gly	stub glycopeptide:charge 1:glycan loss 14	stub glycopeptide:charge 4:glycan loss 6
c-term leu_iso_val_ala	stub glycopeptide:composition ser_thr	stub glycopeptide:charge 1:glycan loss 15	stub glycopeptide:charge 4:glycan loss 7
c-term asn	stub glycopeptide:composition leu_iso_val_ala	stub glycopeptide:charge 1:glycan loss 16	stub glycopeptide:charge 4:glycan loss 8
c-term his	stub glycopeptide:composition asn	stub glycopeptide:charge 1:glycan loss 17	stub glycopeptide:charge 4:glycan loss 9
c-term arg_lys	stub glycopeptide:composition his	stub glycopeptide:charge 1:glycan loss 18	stub glycopeptide:charge 4:glycan loss 10
c-term x	stub glycopeptide:composition arg_lys	stub glycopeptide:charge 1:glycan loss 19	stub glycopeptide:charge 4:glycan loss 11
series b	stub glycopeptide:composition x	stub glycopeptide:charge 1:glycan loss 20	stub glycopeptide:charge 4:glycan loss 12
series y	stub glycopeptide:is_fucosylated 0	stub glycopeptide:charge 2:glycan loss 0	stub glycopeptide:charge 4:glycan loss 13
series stub glycopeptide	stub glycopeptide:is_fucosylated 1	stub glycopeptide:charge 2:glycan loss 1	stub glycopeptide:charge 4:glycan loss 14
unassigned	n-term - 1 pro	stub glycopeptide:charge 2:glycan loss 2	stub glycopeptide:charge 4:glycan loss 15
charge 1	n-term - 1 gly	stub glycopeptide:charge 2:glycan loss 3	stub glycopeptide:charge 4:glycan loss 16
charge 2	n-term - 1 ser_thr	stub glycopeptide:charge 2:glycan loss 4	stub glycopeptide:charge 4:glycan loss 17
charge 3	n-term - 1 leu_iso_val_ala	stub glycopeptide:charge 2:glycan loss 5	stub glycopeptide:charge 4:glycan loss 18
charge 4	n-term - 1 asn	stub glycopeptide:charge 2:glycan loss 6	stub glycopeptide:charge 4:glycan loss 19
charge 5	n-term - 1 his	stub glycopeptide:charge 2:glycan loss 7	stub glycopeptide:charge 4:glycan loss 20
series b:charge 1	n-term - 1 arg_lys	stub glycopeptide:charge 2:glycan loss 8	stub glycopeptide:charge 5:glycan loss 0
series b:charge 2	n-term - 1 x	stub glycopeptide:charge 2:glycan loss 9	stub glycopeptide:charge 5:glycan loss 1
series b:charge 3	n-term - 2 pro	stub glycopeptide:charge 2:glycan loss 10	stub glycopeptide:charge 5:glycan loss 2
series b:charge 4	n-term - 2 gly	stub glycopeptide:charge 2:glycan loss 11	stub glycopeptide:charge 5:glycan loss 3
series b:charge 5	n-term - 2 ser_thr	stub glycopeptide:charge 2:glycan loss 12	stub glycopeptide:charge 5:glycan loss 4
series y:charge 1	n-term - 2 leu_iso_val_ala	stub glycopeptide:charge 2:glycan loss 13	stub glycopeptide:charge 5:glycan loss 5
series y:charge 2	n-term - 2 asn	stub glycopeptide:charge 2:glycan loss 14	stub glycopeptide:charge 5:glycan loss 6
series y:charge 3	n-term - 2 his	stub glycopeptide:charge 2:glycan loss 15	stub glycopeptide:charge 5:glycan loss 7
series y:charge 4	n-term - 2 arg_lys	stub glycopeptide:charge 2:glycan loss 16	stub glycopeptide:charge 5:glycan loss 8
series y:charge 5	n-term - 2 x	stub glycopeptide:charge 2:glycan loss 17	stub glycopeptide:charge 5:glycan loss 9
series stub glycopeptide:charge 1	c-term + 1 pro	stub glycopeptide:charge 2:glycan loss 18	stub glycopeptide:charge 5:glycan loss 10
series stub glycopeptide:charge 2	c-term + 1 gly	stub glycopeptide:charge 2:glycan loss 19	stub glycopeptide:charge 5:glycan loss 11
series stub glycopeptide:charge 3	c-term + 1 ser_thr	stub glycopeptide:charge 2:glycan loss 20	stub glycopeptide:charge 5:glycan loss 12
series stub glycopeptide:charge 4	c-term + 1 leu_iso_val_ala	stub glycopeptide:charge 3:glycan loss 0	stub glycopeptide:charge 5:glycan loss 13
series stub glycopeptide:charge 5	c-term + 1 asn	stub glycopeptide:charge 3:glycan loss 1	stub glycopeptide:charge 5:glycan loss 14
is_glycosylated 0	c-term + 1 his	stub glycopeptide:charge 3:glycan loss 2	stub glycopeptide:charge 5:glycan loss 15
is_glycosylated 1	c-term + 1 arg_lys	stub glycopeptide:charge 3:glycan loss 3	stub glycopeptide:charge 5:glycan loss 16
is_glycosylated 2	c-term + 1 x	stub glycopeptide:charge 3:glycan loss 4	stub glycopeptide:charge 5:glycan loss 17
charge 1:c-term pro	c-term + 2 pro	stub glycopeptide:charge 3:glycan loss 5	stub glycopeptide:charge 5:glycan loss 18
charge 2:c-term pro	c-term + 2 gly	stub glycopeptide:charge 3:glycan loss 6	stub glycopeptide:charge 5:glycan loss 19
charge 3:c-term pro	c-term + 2 ser_thr	stub glycopeptide:charge 3:glycan loss 7	stub glycopeptide:charge 5:glycan loss 20
charge 4:c-term pro	c-term + 2 leu_iso_val_ala	stub glycopeptide:charge 3:glycan loss 8	
charge 5:c-term pro	c-term + 2 asn	stub glycopeptide:charge 3:glycan loss 9	
series:c-term pro b	c-term + 2 his	stub glycopeptide:charge 3:glycan loss 10	
series:c-term pro y	c-term + 2 arg_lys	stub glycopeptide:charge 3:glycan loss 11	
is_glycosylated:c-term pro 0	c-term + 2 x	stub glycopeptide:charge 3:glycan loss 12	
is_glycosylated:c-term pro 1	stub glycopeptide:charge 1:glycan loss 0	stub glycopeptide:charge 3:glycan loss 13	
is_glycosylated:c-term pro 2	stub glycopeptide:charge 1:glycan loss 1	stub glycopeptide:charge 3:glycan loss 14	
stub glycopeptide:is_glycosylated 0	stub glycopeptide:charge 1:glycan loss 2	stub glycopeptide:charge 3:glycan loss 15	
stub glycopeptide:is_glycosylated 1	stub glycopeptide:charge 1:glycan loss 3	stub glycopeptide:charge 3:glycan loss 16	

Table 6: Glycopeptide Intensity Prediction Features. We used these features to model the intensity of peaks associated with a glycopeptide fragmentation event. The ":" symbol denotes an interaction between two or more properties of a fragmentation event. The majority of these features are binary, with the exception of those starting with *stub glycopeptide:composition*, which may take on arbitrary non-negative integer values. *stub glycopeptide* refers to peptide+Y fragments.

2.5 Site-Specific Glycome Network Smoothing

We and others have shown that it is useful to exploit the relationships between biosynthetically nearby glycans when evaluating glycan confidence (Benedetti *et al.*, 2017; Klein *et al.*, 2018). While the previous methods described learning a generalizable model of glycopeptide fragmentation, this method taps into the biological context of a sample, requiring we use tissue-specific and/or disease-specific models. The human serum dataset used the same *N*-glycan neighborhood rules found in Klein *et al.* (2018), while the mouse brain tissue subset used an extended set of neighborhood bounds to take into account biosynthetic pathways absent in humans and other old world primates (Varki, 2017) like the Gal-α-Gal epitope and NeuGc as possible terminal groups.

We extend the method applicable to released glycans we presented in Klein *et al.* (2018) to the glycoforms observed at distinct sites of a glycoprotein, as determined by the high confidence identified glycopeptides spanning those sites, treating each site independently from all others. We generalize the approach to allow us to aggregate information across replicates by defining the variance of a glycan composition to be equal to $\frac{1}{k}$ where k is the number of distinct times that glycan was observed at that site across peptides and across replicates, denoted as

the diagonal variance-covariance matrix $\mathbf{K}$.

We computed the MS1 score s for each glycopeptide passing a joint FDR threshold of 1%, using the same features as in Klein *et al.* (2018), save that we omit the adduct score and set the charge state score to a flat $logit(0.8)$ as the charge state model was not appropriate for glycopeptides. While no MS2-based information is explicitly used to compute s, the 1% FDR threshold enforces that the glycan was observed confidently. We also required each glycan be observed in at least two replicates for it to contribute to network smoothing parameter estimation.

Briefly, the estimation process for a single glycosite begins by constructing a graph $\mathcal{G}$ that spans the glycome database, partitioned in to overlapping neighborhoods with some central tendencies $\boldsymbol{\tau}$. We project our observed glycan confidence estimates $\mathbf{s}$ onto their respective nodes in $\mathcal{G}$. We partition the Laplacian matrix $\mathbf{L}$ of $\mathcal{G}$ into blocks $\begin{bmatrix}\mathbf{L_{oo}} & \mathbf{L_{om}}\\ \mathbf{L_{mo}} & \mathbf{L_{mm}}\end{bmatrix}$ where subscript denotes that the glycan composition along that row/column is observed (o) or missing (m). We also partition a ($|\mathcal{G}|$ by $|\boldsymbol{\tau}|$) neighborhood belonging-ness matrix $\mathbf{A}$'s rows to $\begin{bmatrix}\mathbf{A}_o\\ \mathbf{A}_m\end{bmatrix}$ and $\tau_o = \mathbf{A}_o\tau$, $\tau_m = \mathbf{A}_m\tau$.

We estimated a smoothed version of $\mathbf{s}$, $\boldsymbol{\phi}$, that captures the relatedness of the observed glycans and the neighborhoods of the glycome they come from and propagate this to unobserved glycans, by minimizing the expression:

$$\mathcal{S}(\mathbf{L},\phi,\tau) = \begin{bmatrix}\phi_o - \tau_o\\ \phi_m - \tau_m\end{bmatrix}^t \begin{bmatrix}\mathbf{L_{oo}} & \mathbf{L_{om}}\\ \mathbf{L_{mo}} & \mathbf{L_{mm}}\end{bmatrix} \begin{bmatrix}\phi_o - \tau_o\\ \phi_m - \tau_m\end{bmatrix} \tag{S16}$$

$$\ell = (\mathbf{s} - \boldsymbol{\phi_o})^t\mathbf{K}^{-1}(\mathbf{s} - \boldsymbol{\phi_o}) + \lambda\mathcal{S}(\mathbf{L},\phi,\tau) \tag{S17}$$

For a fixed smoothing factor λ and variance $\mathbf{K}$, we can solve $\boldsymbol{\tau}$ and take the partial derivative w.r.t. ϕ_o:

$$\tau = \mathbf{A}_o\left(0.1\times\mathbf{K} + \frac{1}{\lambda}\mathbf{L}_{oo}^{-1} + \mathbf{A}_o^t\mathbf{A}_o\right)^{-1}\mathbf{s} \tag{S18}$$

$$0 = \frac{\partial\ell}{\partial\phi_o}\left((\mathbf{s} - \boldsymbol{\phi_o})^t\mathbf{K}^{-1}(\mathbf{s} - \boldsymbol{\phi_o}) + \lambda\mathcal{S}(\mathbf{L},\phi,\tau)\right)$$

$$\hat{\phi}_o = \left[\mathbf{K} + \lambda\left(\mathbf{L_{oo}} - \mathbf{L_{om}}\mathbf{L_{mm}^{-1}}\mathbf{L_{mo}}\right)\right]^{-1}(\mathbf{s} - \tau_o) + \tau_o \tag{S19}$$

and then for ϕ_m:

$$0 = \frac{\partial\ell}{\partial\phi_m}\left((\mathbf{s} - \boldsymbol{\phi_o})^t(\mathbf{s} - \boldsymbol{\phi_o}) + \lambda\mathcal{S}(\mathbf{L},\phi,\tau)\right)$$

$$\hat{\phi}_m = -\mathbf{L_{mm}}^{-1}\mathbf{L_{mo}}(\phi_o - \tau_o) + \tau_m \tag{S20}$$

We used grid search to select λ to estimate the network parameters τ, but computed the updated glycan confidence ϕ_o and ϕ_m using $\lambda = 0.2$ or the grid search λ, whichever was lower. Following model fitting, we saved a snapshot of the glycomes annotated with the network smoothed confidence values. To avoid notation ambiguity with the inter-peak reliability model, the symbol ϕ used in Klein *et al.* (2018) to represent the smoothed glycan confidences, we denote the network smoothed glycan confidence $\mathbf{u}$ where $\mathbf{u}_o$ are the observed glycans that contributed to the model and $\mathbf{u}_m$ are the unobserved glycans informed by the model and $\mathbf{u}_o$. We fit a model for each glycosite of each observed protein independently.

When generating a decoy glycan the value u is the same as the target as they are derived from the same glycosylation site on the same protein, however decoy glycans tend to have

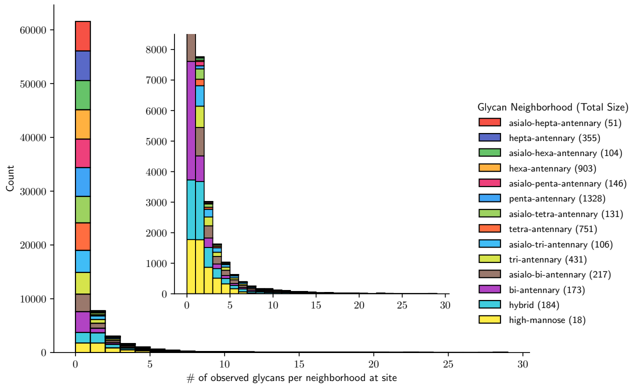

Figure 42: Mouse glycan neighborhood sizes. To contrast Fig. 2i, this histogram counts the number of observed glycans belonging to each neighborhood at each glycosite, with the number of theoretical members in each neighborhood shown in the legend.

poor glycan coverage, reducing the impact that u has on the glycan score, giving smoothed target glycans an advantage. When generating decoy proteins, its glycosite models are identical to the target protein's but reflected to match the reversed protein coordinate of the permuted *N*-glycosylation site. Because decoy peptide matches occur at random, they are expected to happen at random, making it unlikely that they will match with glycans following their site-specific trends, giving target glycopeptides following the expected trend an advantage.

2.6 Glycome Neighborhood Structure

Because when estimating $\boldsymbol{\tau}$, we depend upon the magnitude of values in the corresponding columns of $\mathbf{A}$, a which as described in Klein *et al.* (2018) is a function of neighborhood size and diversity. The larger the neighborhood, the smaller the weight of an individual glycan on that neighborhood in $\mathbf{A}$. Fig 42 shows the number of times a glycosite had been observed with a glycan belonging to each neighborhood. It highlights the abundance of smaller, less mature glycan neighborhoods like high-mannose, and the difficulties faced when estimating parameters for larger neighborhoods which are implicitly less common to begin with. There are many different ways to construct $\mathbf{A}$, and the neighborhood definitions used here were chosen for their generality, though narrower neighborhoods may be more effective in some scenarios.

2.7 Run Time

GlycReSoft is written in Python and Cython/C, compared to C++, C#, or Java, and requires more CPU cycles to do the same amount of work outside of the pure C kernels it wraps. It also does not benefit from shared memory parallelism when distributing work across multiple processors, introducing additional message serialization overhead in addition to the higher cost of process synchronization as opposed to thread synchronization.

GlycReSoft is substantially slower than pGlyco3 when considering purely CPU time costs, because while GlycReSoft may run in a comparable amount of time for a single sample, it consumes many CPUs per sample while pGlyco3 runs a single sample per CPU.

Tool	Phase	Parallelism	Time
GlycReSoft	Preprocessing raw spectra	12	19.46 minutes
	Database construction	5	10.01 minutes
	Database search (Base)	16	9.41 minutes
	Database search (Fragmentation Model)	16	15.96 minutes
	Localization	1	2.28 minutes
	Chromatogram mapping & adduct deconvolution	1	3.72 minutes
	Retention time modeling	4	8.82 minutes
	Serialization	1	9.0 minutes
GlycReSoft	Total (Wall)	-	53.29 minutes
GlycReSoft	Total (CPU)	-	333.85 minutes
pGlyco3	Total	1	81.61 minutes

Table 7: Runtime comparison. Running the base scoring strategy algorithm in GlycReSoft and pGlyco3 on MouseBrain-Z-T-1.raw on an Intel(R) Core(TM) i7-10700K CPU @ 3.80GHz with 16 cores and 64 GB of RAM Windows 10 workstation. The full study was executed on a shared computing cluster running AlmaLinux and using a heterogenous mixture of CPU nodes, which may introduce slight variations in results due to differences in floating point rounding, running times, and shared resources. Totals for GlycReSoft were computed using "Base" scoring model, but the added runtime cost of the "Fragmentation Model" does not substantially change the runtime of subsequent downstream operations beyond the the database search step.

2.8 Revision Counts

The retention time revision process described in section 2.1 operates on chromatographic features, which in turn broadcast updates to all MS2 spectra which it contains, provided they satisfy the requirements described previously. Table 8 shows that there can be large variations in revisions from tissue to tissue in the mouse dataset, and finding a stable solution with the added isobaric substitution ambiguities of NeuAc+Hex vs NeuGc+dHex as well as ammonium adduction likely made things even more difficult to deconvolve. Table 9 shows a higher average update rate compared to the mouse tissue dataset, but whether this was caused by differences in sample preparation/handling, reduced complexity of the glycome with substitutions to consider, or other factor is unknown.

Our prior work in Klein and Zaia (2020) suggests a larger fraction of the glycopeptides detected could be mis-assigned, but differences in chromatography and sample composition make comparisons difficult. Samples in which a small number of glycosites are analyzed in detail are much easier for our relative retention time technique, compared to serum or tissue digests with many, many glycosites at varying degrees of depth.

An extension of this method might be reasonable using an additional pass where all glycopeptides' absolute retention times have their glycan compositions' contributions deducted, producing one or more measures of retention time for the same peptide backbone sequence, followed by either fitting or fine-tuning a peptide retention time model. This peptide retention time model would then be able to provide a reference point for all peptide backbone sequences, although such a construction compounds modeling error at least twice, and the peptide retention time model may not provide compatible confidence interval estimation techniques.

Search<br>Sample	Base<br>Count	Percentage	Fragment Model<br>Count	Percentage	Fragment Model + Smoothed<br>Count	Percentage	Smoothed<br>Count	Percentage
MouseBrain-Z-T-1	112	2.90%	152	3.76%	152	3.70%	119	2.89%
MouseBrain-Z-T-2	202	5.70%	215	5.96%	216	5.94%	202	5.57%
MouseBrain-Z-T-3	140	3.94%	196	5.13%	201	5.14%	166	4.39%
MouseBrain-Z-T-4	134	3.56%	182	4.68%	174	4.35%	143	3.58%
MouseBrain-Z-T-5	126	3.27%	181	4.63%	185	4.67%	147	3.68%
MouseHeart-Z-T-1	63	2.98%	66	3.09%	68	3.11%	62	2.84%
MouseHeart-Z-T-2	35	2.44%	36	2.36%	38	2.45%	40	2.62%
MouseHeart-Z-T-3	44	2.73%	50	3.08%	49	2.87%	49	2.94%
MouseHeart-Z-T-4	70	4.17%	68	3.93%	62	3.46%	66	3.84%
MouseHeart-Z-T-5	46	3.36%	42	2.90%	44	2.85%	45	3.07%
MouseKidney-Z-T-1	166	3.40%	185	3.81%	187	3.78%	156	3.10%
MouseKidney-Z-T-2	186	3.86%	205	4.20%	199	4.03%	195	3.91%
MouseKidney-Z-T-3	200	4.03%	207	4.19%	203	4.04%	197	3.85%
MouseKidney-Z-T-4	161	3.25%	193	3.90%	193	3.81%	139	2.77%
MouseKidney-Z-T-5	207	4.05%	215	4.18%	208	4.00%	185	3.52%
MouseLiver-Z-T-1	34	1.06%	40	1.24%	42	1.25%	42	1.23%
MouseLiver-Z-T-2	19	0.67%	20	0.68%	21	0.70%	20	0.67%
MouseLiver-Z-T-3	43	1.30%	47	1.38%	49	1.40%	39	1.11%
MouseLiver-Z-T-4	23	0.79%	33	1.12%	31	1.01%	28	0.92%
MouseLiver-Z-T-5	32	1.02%	33	1.01%	32	0.96%	30	0.90%
MouseLung-Z-T-1	70	1.99%	83	2.25%	78	2.07%	75	2.02%
MouseLung-Z-T-2	78	2.06%	93	2.43%	91	2.31%	86	2.17%
MouseLung-Z-T-3	86	2.27%	90	2.29%	91	2.26%	87	2.20%
MouseLung-Z-T-4	71	1.91%	73	1.93%	73	1.89%	66	1.72%
MouseLung-Z-T-5	92	2.84%	98	2.97%	93	2.73%	93	2.73%

Table 8: Mouse tissue dataset revisions from RT modeling. The number of revised glycopeptide chromatographic features in each sample in each search for each scoring method in the mouse tissue dataset. These correspond to MS1 features, not MSn spectra.

Search	Base		Fragment Model		Fragment Model + Smoothed		Smoothed	
Sample	Count	Percentage	Count	Percentage	Count	Percentage	Count	Percentage
RB_161102_JANUS39_TIO2.db	93	6.22%	98	6.36%	101	6.47%	85	5.50%
RB_161104_Janus_10_TiO2.db	79	6.34%	73	5.58%	75	5.62%	72	5.54%
RB_161104_Janus_20_TiO2.db	90	6.61%	87	6.29%	96	6.78%	84	5.97%
RB_161104_Janus_21_TiO2.db	78	6.20%	79	6.10%	82	6.18%	76	5.68%
RB_161104_Janus_36_TiO2.db	71	5.24%	71	5.27%	72	5.27%	72	5.05%
RB_161104_Janus_40_TiO2.db	96	6.71%	89	6.03%	97	6.52%	90	5.85%
RB_161104_Janus_41_TiO2.db	51	5.30%	65	6.53%	64	6.34%	52	5.19%
RB_161104_Janus_45_TiO2.db	72	5.99%	72	5.91%	73	5.86%	65	5.02%
RB_161104_Janus_4_TiO2.db	50	4.19%	58	4.72%	58	4.63%	46	3.87%
RB_161104_Janus_51_TiO2.db	80	5.91%	73	5.20%	74	5.20%	64	4.46%
RB_161104_Janus_53_TiO2.db	85	6.12%	88	6.16%	88	6.09%	81	5.41%
RB_161104_Janus_58_TiO2.db	83	5.50%	83	5.39%	82	5.23%	80	5.11%
RB_161104_Janus_59_TiO2.db	70	6.19%	68	5.83%	67	5.66%	67	5.44%
RB_161104_Janus_5_TiO2.db	65	5.39%	65	5.20%	65	5.18%	61	4.96%
RB_161104_Janus_63_Tio2.db	85	5.52%	86	5.48%	92	5.76%	85	5.23%
RB_161104_Janus_65_TiO2.db	75	5.91%	77	5.91%	79	5.98%	74	5.70%
RB_161104_Janus_66_TiO2.db	77	5.83%	79	5.89%	77	5.57%	77	5.56%
RB_161104_Janus_67_TiO2.db	70	5.11%	71	5.06%	74	5.25%	69	4.87%
RB_161104_Janus_69_TiO2.db	74	5.86%	82	6.39%	82	6.31%	72	5.45%
RB_161104_Janus_70_TiO2.db	88	6.25%	89	6.21%	90	6.19%	82	5.60%
RB_161104_Janus_74_TiO2.db	79	6.00%	79	5.81%	82	5.98%	75	5.43%
RB_161104_Janus_80_TiO2.db	83	6.02%	77	5.55%	78	5.55%	78	5.29%
RB_161104_Janus_85_TiO2.db	66	4.85%	67	4.83%	70	5.00%	60	4.31%
RB_161104_Janus_9_TiO2.db	69	5.15%	74	5.49%	75	5.48%	68	4.85%

Table 9: Human serum dataset revisions from RT modeling. The number of revised glycopeptide chromatographic features in each sample for the base scoring method in the PXD005931. These correspond to MS1 features, not MSn spectra.

References

Aboufazeli, F. and Dodds, E. D. (2018). Precursor Ion Survival Energies of Protonated N-Glycopeptides and their Weak Dependencies on High Mannose N-Glycan Composition in Collision-Induced Dissociation. *The Analyst*, pages 4459–4468.

Benedetti, E., Pučić-Baković, M., Keser, T., Wahl, A., Hassinen, A., Yang, J. Y., Liu, L., Trbojević-Akmačić, I., Razdorov, G., Štambuk, J., Klarić, L., Ugrina, I., Selman, M. H., Wuhrer, M., Rudan, I., Polasek, O., Hayward, C., Grallert, H., Strauch, K., Peters, A., Meitinger, T., Gieger, C., Vilaj, M., Boons, G. J., Moremen, K. W., Ovchinnikova, T., Bovin, N., Kellokumpu, S., Theis, F. J., Lauc, G., and Krumsiek, J. (2017). Network inference from glycoproteomics data reveals new reactions in the IgG glycosylation pathway. *Nature Communications*, **8**(1), 1–15.

Frank, A. M. (2009). Predicting intensity ranks of peptide fragment ions. *Journal of proteome research*, **8**(5), 2226–40.

Gabriels, R., Martens, L., and Degroeve, S. (2019). Updated MS²PIP web server delivers fast and accurate MS² peak intensity prediction for multiple fragmentation methods, instruments and labeling techniques. *Nucleic Acids Research*, pages 1–5.

Klein, J. and Zaia, J. (2020). Relative Retention Time Estimation Improves N-Glycopeptide Identifications by LC–MS/MS. *Journal of Proteome Research*, **19**(5), 2113–2121.

Klein, J., Carvalho, L., and Zaia, J. (2018). Application of network smoothing to glycan LC-MS profiling. *Bioinformatics*, **34**(20), 3511–3518.

Kolli, V., Roth, H. A., De La Cruz, G., Fernando, G. S., and Dodds, E. D. (2015). The role of proton mobility in determining the energy-resolved vibrational activation/dissociation channels of N-glycopeptide ions. *Analytica Chimica Acta*, **896**, 85–92.

Varki, A. (2017). Biological roles of glycans. *Glycobiology*, **27**(1), 3–49.

Zeng, W. F., Cao, W. Q., Liu, M. Q., He, S. M., and Yang, P. Y. (2021). Precise, fast and comprehensive analysis of intact glycopeptides and modified glycans with pGlyco3. *Nature Methods*, **18**(12), 1515–1523.

Zubarev, R. A., Zubarev, A. R., and Savitski, M. M. (2008). Electron Capture/Transfer versus Collisionally Activated/Induced Dissociations: Solo or Duet? *Journal of the American Society for Mass Spectrometry*, **19**(6), 753–761.
